# Increased Phospholipid Flux Bypasses Overlapping Essential Requirements for the Yeast Sac1p Phosphoinositide Phosphatase and ER-PM Membrane Contact Sites

**DOI:** 10.1016/j.jbc.2023.105092

**Published:** 2023-07-26

**Authors:** Aleksa Nenadic, Mohammad F. Zaman, Jesper Johansen, Matthew W. Volpiana, Christopher T. Beh

**Affiliations:** 1 Department of Molecular Biology and Biochemistry, Simon Fraser University, Burnaby, British Columbia, Canada; 2 Centre for Cell Biology, Development, and Disease, Simon Fraser University, Burnaby, British Columbia, Canada

**Keywords:** autophagy, endoplasmic reticulum (ER), ER-PM membrane contact sites, phosphatidylserine, phosphatidylinositol phosphate phosphatase, phosphoinositides, phospholipid metabolism, plasma membrane (PM), *Saccharomyces cerevisiae*

## Abstract

In budding yeast cells, much of the inner surface of the plasma membrane (PM) is covered with the endoplasmic reticulum (ER). This association is mediated by seven ER membrane proteins that confer cortical ER-PM association at membrane contact sites (MCSs). Several of these membrane “tether” proteins are known to physically interact with the phosphoinositide phosphatase Sac1p. However, it is unclear how or if these interactions are necessary for their interdependent functions. We find that *SAC1* inactivation in cells lacking the homologous synaptojanin-like genes *INP52* and *INP53* results in a significant increase in cortical ER-PM MCSs. We show in *sac1*Δ, *sac1*^ts^*inp52*Δ *inp53*Δ, or Δ-super-tether (Δ-s-tether) cells lacking all seven ER-PM tethering genes that phospholipid biosynthesis is disrupted and phosphoinositide distribution is altered. Furthermore, *SAC1* deletion in Δ-s-tether cells results in lethality, indicating a functional overlap between *SAC1* and ER-PM tethering genes. Transcriptomic profiling indicates that *SAC1* inactivation in either Δ-s-tether or *inp52*Δ *inp53*Δ cells induces an ER membrane stress response and elicits phosphoinositide-dependent changes in expression of autophagy genes. In addition, by isolating high-copy suppressors that rescue *sac1*Δ Δ–s-tether lethality, we find that key phospholipid biosynthesis genes bypass the overlapping function of *SAC1* and ER-PM tethers and that overexpression of the phosphatidylserine/phosphatidylinositol-4-phosphate transfer protein Osh6 also provides limited suppression. Combined with lipidomic analysis and determinations of intracellular phospholipid distributions, these results suggest that Sac1p and ER phospholipid flux controls lipid distribution to drive Osh6p-dependent phosphatidylserine/phosphatidylinositol-4-phosphate counter-exchange at ER-PM MCSs.

The endoplasmic reticulum (ER) represents the major source of lipid biosynthesis within eukaryotic cells, and the plasma membrane (PM) is the primary destination for many of those lipids. Some lipids are transported to and from the PM by vesicular transport, but significant amounts are transferred by nonvesicular mechanisms, which are facilitated by membrane association at and near ER-PM membrane contact sites (MCSs) where the two membranes are affixed (1, 2, 3, 4). ER-PM MCSs serve not only as an interface for the direct exchange of lipids but also as a nexus to coordinate lipid production with the regulation of PM composition and its expansion during cell growth (5, 6).

In budding yeast, roughly half of the PM is associated with the ER (2, 5, 7). Membrane association is conferred by “primary” membrane tether proteins that are both necessary and sufficient for ER-PM contact under standard growth conditions (3, 5, 6). These tether proteins staple sections of cortical ER (cER) to the PM to generate MCSs. Six primary tethers are conserved proteins, while another represents a yeast-specific tethering factor (7, 8, 9, 10). All these tethering factors are ER-integral membrane proteins that individually, or with other interacting proteins, reach across from the cER to contact the PM (6). The conserved tethers include (i) the yeast homologues of vesicle-associated membrane protein–associated protein, Scs2p and Scs22p; (ii) the Extended Synaptotagmins (E-Syts), Tcb1p-3p; and (iii) Ist2p, which is a member of the TMEM16/Anoctamin family of ion channels and phospholipid scramblases (8, 9, 11, 12). Ice2p represents a yeast-specific factor that confers ER attachments with both the PM and lipid droplets (12, 13). In dividing cells, Ice2p links cER to the PM to facilitate cER movement along the PM from yeast mother cells into daughter cells (12, 13). During stationary phase, however, Ice2p confers ER-lipid droplet association (14). Elimination of *ICE2* along with the conserved tether protein genes generates so-called Δ-super-tether (Δ-s-tether) cells (*tcb1*Δ *tcb2*Δ *tcb3*Δ *scs2*Δ *scs22*Δ *ist2*Δ *ice2*Δ) and reduces ER-PM association from 48% to 1.7%, which is below calculated levels of stochastic association between the ER and PM (5). Elimination of these tethers imparts a moderate cell growth defect that can be rescued by supplementing cell cultures with choline, a precursor of phospholipid biosynthesis, although choline treatment does not reestablish ER-PM MCSs (5). These results suggest that yeast ER-PM MCSs have an important role in regulating phospholipid metabolism.

The synthesis of many membrane lipids involves phosphatidic acid (PA) as an initial precursor (Fig. 1*A*) (15, 16, 17). In yeast cells, PA is generated from diacylglycerol (DAG) in the PM, or lysophosphatidic acid in the ER (18, 19, 20, 21, 22). In the “CDP-DAG pathway,” PA is converted to cytidine diphosphate–diacylglycerol (CDP-DAG) *via* CDP-DAG synthase, encoded by *CDS1* (23). CDP-DAG is then required for phosphatidylserine (PS) synthesis, which in turn is a precursor for phosphatidylethanolamine (PE) and phosphatidylcholine (PC) production (24, 25, 26, 27, 28, 29). PE and PC can also be generated through the alternate “Kennedy pathway” that utilizes exogenous ethanolamine or choline as precursors (30, 31, 32, 33, 34, 35, 36, 37). Together with inositol, CDP-DAG also forms phosphatidylinositol (PI), which is the precursor for all phosphoinositides, including phosphatidylinositol-4-phosphate (PI4P) and additional phosphorylated forms like phosphatidylinositol-4,5-bisphosphate (PI(4,5)P_2_) (38, 39, 40, 41, 42, 43). In yeast, PI is also precursor for complex sphingolipid biosynthesis in which PI is coupled with ceramide to generate inositol phosphoryl-ceramide (IPC), mannose-inositol phosphorylceramide, and mannose (inositol-P)2-phosphorylceramide (44, 45, 46, 47). As a starting point for phospholipid synthesis, the production of PA is pivotal in regulating levels of phospholipids, sphingolipids, and general membrane composition.

**Figure 1:**
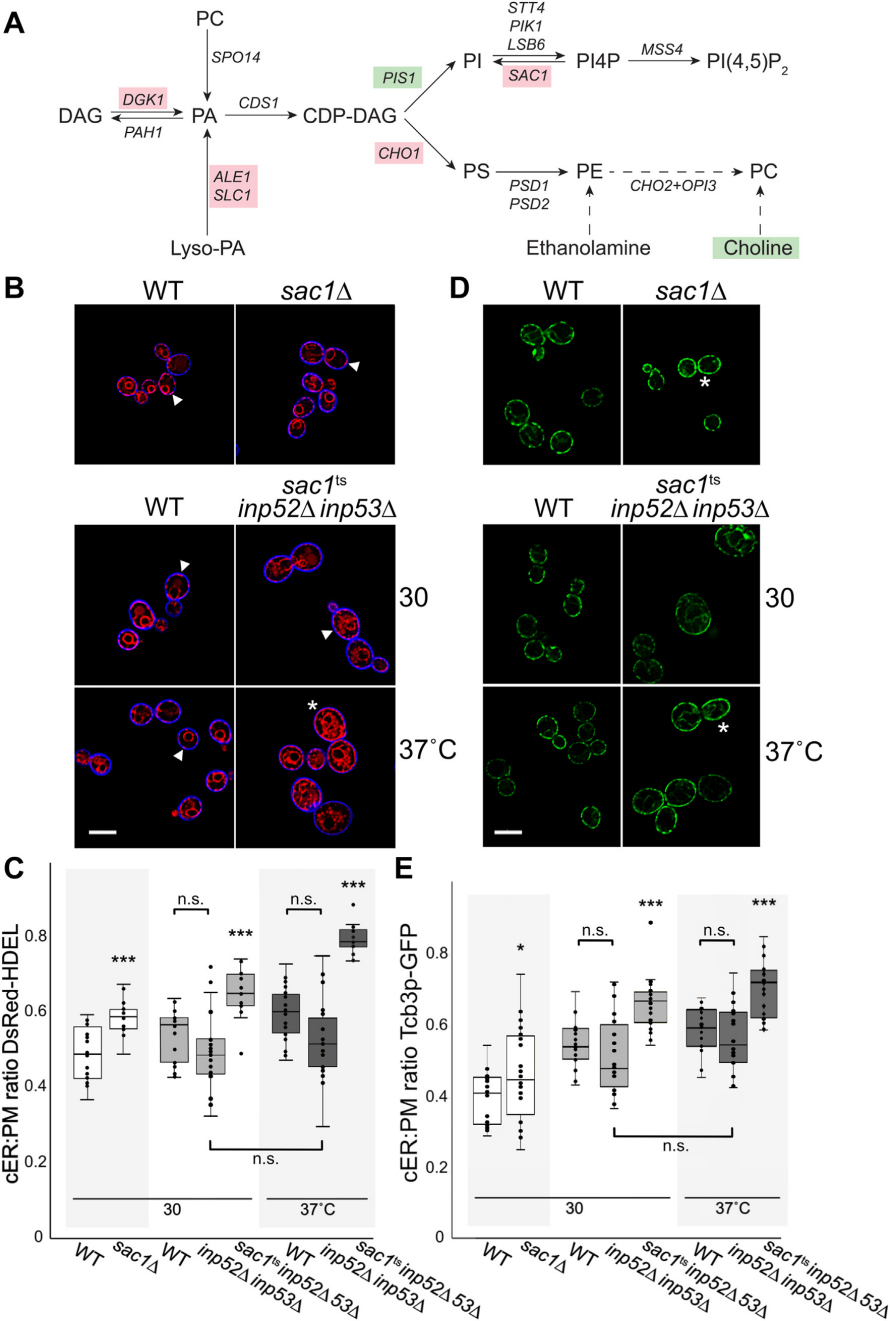
**PI phosphate phosphatases negatively affect ER-PM association.***A*, schematic depicting phospholipid biosynthesis pathway in *Saccharomyces cerevisiae*. Genes representing each enzymatic step are shown as indicated, where genes highlighted in *pink* represent dosage suppressors of *sac1*Δ Δ-s-tether cells identified in this study and those in *green* did not suppress (including choline). *B*, representative images of *sac1*Δ (CBY2809) cells with their WT control (BY4741), and *sac1*^ts^*inp52*Δ *inp53*Δ (AAY143) cells with their WT control (SEY6210), expressing the ER marker DsRed-HDEL (pRS416-DsRed-HDEL) and stained with a blue cell surface dye (MemBrite Fix 405/430). Cells were incubated either at 30 °C or at 37 °C for 1 h, as indicated. *C*, ratios of cER coverage per total distance of each cell perimeter/PM corresponding to (*B*) (N ≥ 20 cells/strain). *D*, representative images of Tcb3p-GFP (pWK092) expressed in *sac1*Δ (CBY2809) cells and WT (BY4741) control, and *sac1*^ts^*inp52*Δ *inp53*Δ (AAY143) cells and WT (SEY6210) control cells, incubated either at 30 °C or at 37 °C for 1 h. *E*, ratios of Tcb3p-GFP-fluorescent cER coverage per total distance of the cell perimeter/PM corresponding to (*D*) (N ≥ 20 cells/strain). Arrowheads indicate examples of cER associated with the PM, and asterisks indicate cells with nearly absolute coverage of the cortex with ER. The scale bar represents 5 μm. ∗*p* < 0.003; ∗∗∗*p* < 0.00015. CDP, cytidine diphosphate; cER, cortical endoplasmic reticulum; DAG, diacylglycerol; ER, endoplasmic reticulum; PA, phosphatidic acid; PC, phosphatidylcholine; PE, phosphatidylethanolamine; PI, phosphatidylinositol; PI(4,5)P_2_, phosphatidylinositol-4,5-bisphosphate; PI4P, phosphatidylinositol-4-phosphate; PM, plasma membrane; PS, phosphatidylserine.

In addition to defects in phospholipid biosynthesis, ER-PM MCSs also impact phosphoinositide distribution within cell membranes (5, 7, 48, 49, 50). In yeast, PI4P is an essential lipid that is predominantly localized in the Golgi and exocytic vesicles and in the PM at sites of polarization within budding daughter cells (48, 51, 52). In the absence of ER-PM MCSs, PI4P distribution spreads throughout the PM in both mother cells and daughter buds (5). This PI4P mislocalization throughout the PM is also detected when *SAC1* is deleted (48). *SAC1* encodes an ER-localized PI4P phosphatase that dephosphorylates PI4P producing PI (53). Because of the similar defects in PI4P membrane distribution, and because most ER-PM tether proteins physically interact with Sac1p, it is proposed that Sac1p acts at ER-PM MCSs to regulate PI4P levels in both membranes (5, 7, 48, 54). Although *SAC1* is not essential for yeast growth, deletion of *SAC1* in Δ-s-tether cells is lethal, which confirms a functional interaction (5).

Here, we analyze how Sac1p and ER-PM tether proteins cooperate to regulate phospholipid biosynthesis and lipid distribution through the generation of a conditionally lethal *sac1*^*ts*^ Δ-s-tether mutant. In this mutant, the combined requirements for *SAC1* and ER-PM MCSs to maintain ER morphology, cellular lipid composition, and lipid distributions were tested. Lipidomic analysis indicated that phospholipid biosynthesis is disrupted in *sac1*^*ts*^ Δ-s-tether cells and the membrane distributions of specific phospholipids and phosphoinositides are disrupted. Effectors of the overlapping functions of *SAC1* and ER-PM MCS were also identified through the isolation of high-copy suppressors of *sac1*^*ts*^ Δ-s-tether lethality. Several CDP-DAG pathway genes that promote the flux of phospholipid synthesis producing PS are bypass suppressors of *sac1*Δ Δ-s-tether lethality. These suppressors do not restore normal cER-PM contact, and ER-PM tethering was found to be specifically dependent on established ER-PM tethers. The transcriptomic profile of *sac1*^*ts*^ Δ-s-tether cells indicates that the combined effect of these mutations results in membrane stress and phosphoinositide-dependent autophagy dysregulation. This study reveals that ER-PM MCSs maintain cellular membrane lipid distribution by regulating phospholipid flux, contributing to PS and PI4P exchange between the ER and PM thereby generally affecting phosphoinositide distribution and homeostasis.

## Results

### Eliminating PI phosphate phosphatases increases cER-PM contact

In yeast, the ER-localized PI phosphate phosphatase Sac1p turns over PI4P transferred from the PM and the Golgi (54). In most *Saccharomyces cerevisiae* strains, *SAC1* is not an essential gene unless its deletion is combined with mutations in other homologous PI phosphate phosphatases (*i.e.*, *INP52* and *INP53*) (55). Sac1p also physically interacts with most primary ER-PM tether proteins, which are all ER integral membrane proteins (7). Although the elimination of these ER-PM tether proteins is not lethal, Δ-s-tether cells lacking tethers are inviable if *SAC1* is also deleted (5). Based on these genetic results we hypothesized that Sac1p and ER-PM tethers function in parallel but independent pathways, despite that Sac1p physically interacts with many ER-PM tethers. Sac1p can act in *trans* by dephosphorylating PI4P on closely apposed membranes *in vitro*, but the genetic interaction between *SAC1* and the ER-PM tether genes is also consistent with a model in which Sac1p acts in *cis* on PI4P transferred to the ER from the PM *via* ER-PM MCSs (48, 54). It is also unknown if these functional interactions between Sac1p and ER-PM tethers has any regulatory impact on the assembly of cER-PM contact sites.

To determine if Sac1p affects cER-PM association, confocal microscopy was used to analyze the juxtaposition of the ER marker DsRed-HDEL relative to the MemBrite-stained cell cortex in *sac1*Δ cells. As compared with congenic wildtype (WT) cells where 51% of the inner surface of the PM is covered with cER, cER-PM association increases to 59% in *sac1*Δ cells (Fig. 1, *B* and *C*). Because the essential activity of Sac1p overlaps with the other yeast PI phosphate phosphatases, namely, Inp52p and Inp53p, we investigated the combined effect of PI phosphate phosphatase inactivation using the temperature conditional *sac1-23*^ts^ allele in cells lacking *INP52* and *INP53*. The mutations that define the *sac1-23*^ts^ allele lie within and closely adjacent to the catalytic Sac phosphatase domain of Sac1p, and at elevated temperatures *sac1-23*^ts^ mutant cells cannot turn over PI4P in the PM (55). At 37 °C for 1 h, the area of the PM covered with cER increases to 79% in temperature-sensitive *sac1-23*^*ts*^ *inp52*Δ *inp53*Δ cells, relative to 60% in its congenic WT control strain (Fig. 1, *B* and *C*). At 30 °C, a temperature at which *sac1-23*^ts^ is at least partially functional, 65% of the PM is covered with cER in *sac1*^*ts*^ *inp52*Δ *inp53*Δ cells, which still represents a statistically significant increase compared with the congenic WT control. Regardless of temperature, the *inp52*Δ *inp53*Δ mutations by themselves have no effect on cER-PM association as compared with WT (Fig. 1*C*). Although *SAC1* deletion leads to moderate increases in cER-PM association, eliminating overlapping activities of the three homologous PI4P phosphatases further spreads cER along the PM, likely due to increased MCSs.

The dsRed-HDEL marker shows general ER morphology, including specific and nonspecific associations of cER with the PM, whereas Tcb3p-GFP is a marker that specifically identifies direct tether attachment sites between the cER and the PM (4). Thus, as a direct measure of membrane contact, we examined the Tcb3p-GFP ER-PM MCSs that are known to proliferate in response to membrane stress and specific lipid transfer mutants (50). In WT cells, Tcb3p-GFP fluorescence was detected on average covering 40% of the PM and the deletion of *SAC1* results in a modest increase of Tcb3p MCSs to 46% (Fig. 1, *D* and *E*). The slight increase in Tcb3p ER-PM MCSs in *sac1*Δ cells, however, is consistent with the observed moderate increase in cER-PM association.

To determine if eliminating multiple PI phosphate phosphatases affects the spread of Tcb3p ER-PM MCSs, the cortical localization of the Tcb3p-GFP ER-PM tether was analyzed in *sac1*^*ts*^ *inp52*Δ *inp53*Δ cells at 30 and 37 °C (Fig. 1, *D* and *E*). In *sac1*^*ts*^ *inp52*Δ *inp53*Δ cells cultured at the permissive growth temperature of 30 °C, cortical coverage of Tcb3p-GFP increases to a mean of 66%, up from 55% in the congenic WT strain (Fig. 1, *D* and *E*). After 1 h at 37 °C, however, Tcb3p-GFP fluorescence in *sac1*^*ts*^ *inp52*Δ *inp53*Δ cells climbs to 70% of the cortical surface of the PM, as compared with 58% in the congenic WT control under the same conditions (Fig. 1*E*). Regardless of temperature, in *inp52*Δ *inp53*Δ cells no significant change in cortical Tcb3p-GFP is detected, relative to WT (Fig. 1*E*). These results indicate that all three PI phosphate phosphatases contribute to Tcb3p regulation of cER-PM association, where *SAC1* by itself has a modest but otherwise redundant role. After multiple attempts to delete *TCB3* in *sac1*^*ts*^ *inp52*Δ *inp53*Δ cells, we were unable to obtain viable *sac1*^*ts*^ *inp52*Δ *inp53*Δ *tcb3*Δ transformants and could not directly test if Tcb3p alone is essential for the observed increases in cER-PM association.

**Figure 2:**
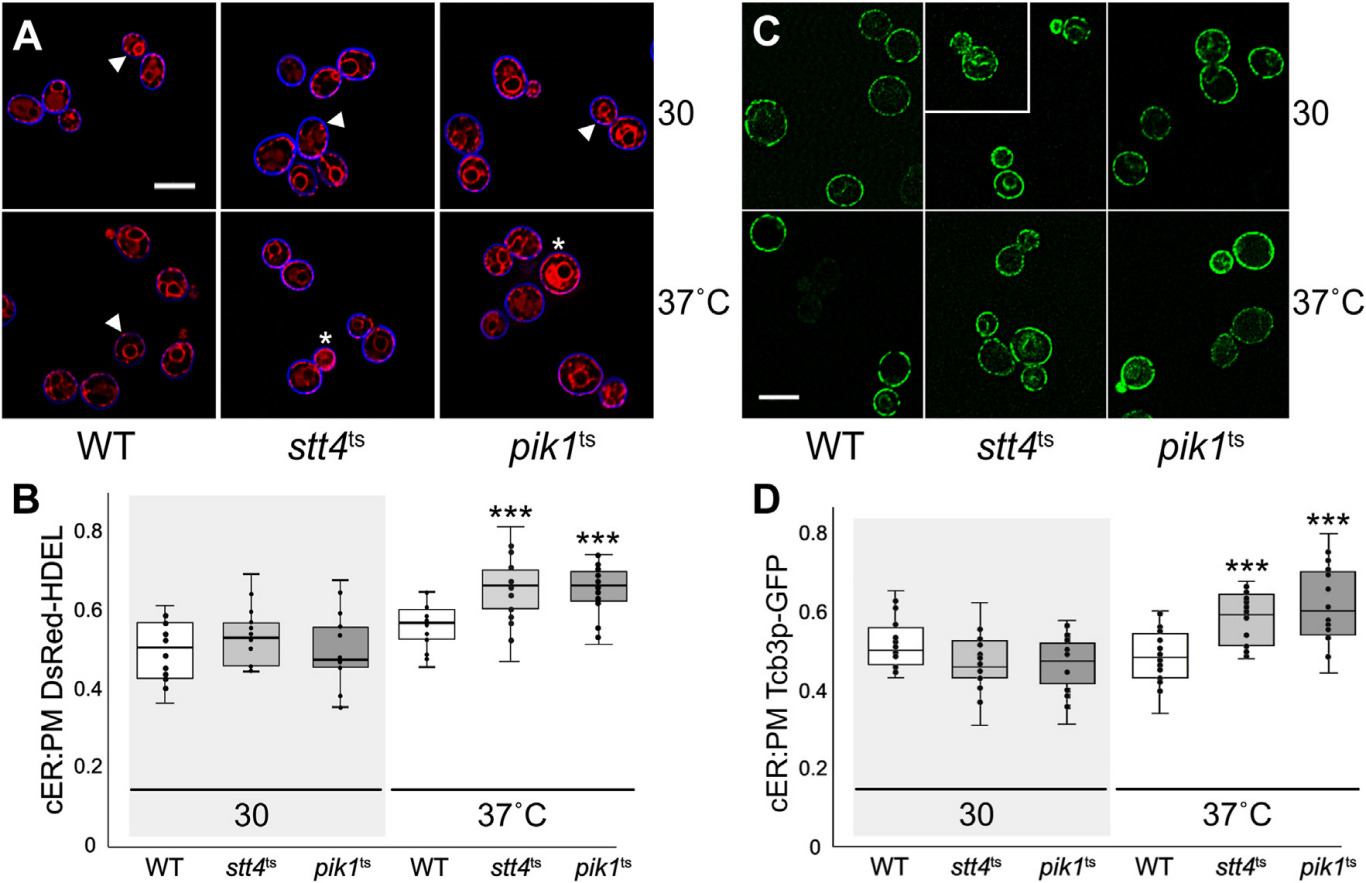
**Inactivation of the phosphatidylinositol kinases Pik1p or Stt4p increases cER-PM association.***A*, representative images of WT (BY4741), *stt4*^ts^ (CBY5090), or *pik1*^ts^ (CBY5092) cells expressing the ER marker DsRed-HDEL (pRS416-DsRed-HDEL) and stained with a blue cell surface dye (MemBrite Fix 405/430). Cells were incubated at either 30 °C or 37 °C for 1 h, as indicated. *B*, ratios of cER coverage per total perimeter distance of the PM corresponding to (*A*) (N = 20 cells/strain). *C*, representative images of Tcb3p-GFP (pWK092) expressed in WT, *stt4*^ts^ or *pik1*^t^ cells, incubated at either 30 °C or 37 °C for 1 h. *D*, ratios of Tcb3p-associated cER coverage per total distance of the cell perimeter/PM corresponding to (*C*) (N = 20 cells/strain). *Arrowheads* indicate cER associated with the PM, and *asterisks* indicate cells with nearly absolute coverage of the cortex with ER. The scale bar represents 5 μm. ∗∗∗*p* ≤ 0.00015. cER, cortical endoplasmic reticulum; ER, endoplasmic reticulum; PM, plasma membrane.

The PI-4-kinases Stt4p and Pik1p generate PI4P from PI within the PM and Golgi, respectively, and these PI-4-kinases represent opposing activities to Sac1p and the other PI4P phosphatases (55, 56). If ER-PM MCS formation is directly affected by cellular PI4P levels, then *stt4*^ts^ and/or *pik1*^ts^ mutants are predicted to have the opposite effect on ER-PM association as observed in *sac1*Δ cells. However, in both *stt4*^ts^ and *pik1*^t^ cells, cortical dsRed-HDEL increases to 66% along the PM relative to 57% in WT cells, after 1 h at 37 °C (Fig. 2, *A* and *B*). Concomitant with the increase in cER association with the PM, Tcb3p ER-PM contact sites also proliferate along the PM. In both *stt4*^ts^ and *pik1*^t^ cells, cortical Tcb3p-GFP fluorescence increases to 58 and 62%, respectively, as compared with 49% observed in the congenic WT control at 37 °C (Fig. 2, *C* and *D*). Because any change in PI4P metabolism increases ER-PM association, ER-PM MCS assembly is generally induced by phosphoinositide dysregulation. Although it is unclear how PI4P homeostasis affects ER-PM MCSs, it is clear that disruption of *SAC1* or other PI4P regulators increases ER-PM MCSs as shown by increased Tcb3p expression at the cell cortex.

### The lethal combination of *SAC1* mutations in cells lacking ER-PM MCSs disrupts phospholipid and sphingolipid metabolism

**Figure 3:**
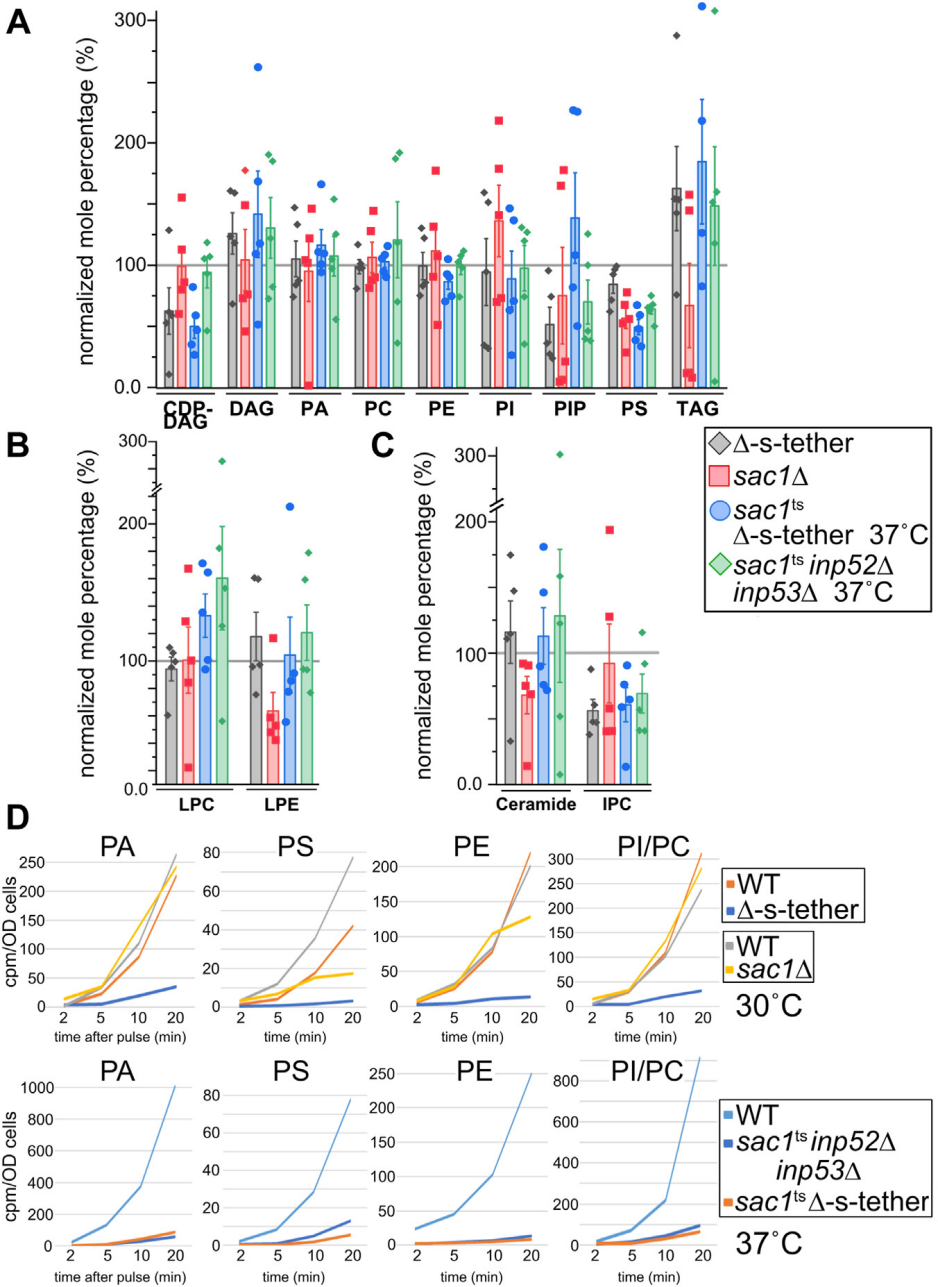
**Lipidomic profiles of *sac1*Δ, *sac1***^**ts**^***inp52*Δ *inp53*Δ, Δ-s-tether, and *sac1***^**ts**^**Δ-s-tether cells exhibit defects in phospholipid and sphingolipid biosynthesis.***A*, phospholipid composition of *sac1*Δ (CBY2809) and Δ-s-tether (CBY5838) at 30 °C, and *sac1*^ts^*inp52*Δ *inp53*Δ (AAY143) and *sac1*^ts^ Δ-s-tether (CBY6345) cells at 37 °C, as a normalized mole percentage relative to WT (SEY6210) (set as 100%) cultured under the same conditions. *B*, lysophospholipid composition of the mutants in (*A*), as a normalized mole percentage relative to WT. *C*, ceramide and IPC composition of the mutants in (*A*), as a normalized mole percentage relative to WT. The lipidomics data represent the mean ± SEM derived from five independent samples as shown. *D*, pulse labeling analysis of phospholipid synthetic flux in WT, *sac1*Δ, and Δ-s-tether at 30 °C, as well as in WT, *sac1*^ts^*inp52*Δ *inp53*Δ, and *sac1*^ts^ Δ-s-tether cells at 37 °C. Glycerophospholipids were extracted 2, 5, 10, and 20 min after addition of ^32^P to log-phase cells, and lipids were separated *via* thin layer chromatography and quantified. In WT, label was first incorporated into PA followed by labeling of PE, PS, and PI/PC observed in varying amounts; mutant cells exhibited reduced levels of label incorporation into phospholipids. PI and PC could not be adequately resolved and are presented as a collective measurement. Each time point for each strain represents the average of duplicate independent analyses. CDP, cytidine diphosphate; DAG, diacylglycerol; IPC, inositol phosphoryl-ceramide; LPC, lysophosphatidylcholine; LPE, lysophosphatidylethanolamine; PA, phosphatidic acid; PC, phosphatidylcholine; PE, phosphatidylethanolamine; PI, phosphatidylinositol; PIP, phosphatidylinositol phosphate; PS, phosphatidylserine; TAG, triacylglycerol.

Lipidomic assays revealed significant defects in the regulation of phospholipid biosynthesis in Δ-s-tether cells (5). We hypothesized that the lethality of *SAC1* deletion in Δ-s-tether might be caused by further exacerbation of this lipid dysregulation. To perform lipidomic analysis on cells lacking both ER-PM tethers and *SAC1*, a temperature-conditional *sac1*^*ts*^ Δ-s-tether strain was generated by transforming a plasmid containing the *sac1-23*^ts^ mutation into *sac1*Δ Δ-s-tether cells, which are otherwise inviable. After incubation at 37 °C for 1 h, lipidomic analysis revealed significant differences in lipid composition between *sac1*^*ts*^ Δ-s-tether, Δ-s-tether, and *sac1*^*ts*^ *inp52*Δ *inp53*Δ strains, as normalized to WT (Fig. 3). In agreement with previous reports, in Δ-s-tether cells many phospholipid levels are significantly reduced including PS and CDP-DAG (Fig. 3*A*) (5). These reductions are likely due to defects in the biosynthetic utilization of the phospholipid precursor DAG, as indicated by its increased levels and the significant accumulation of triacylglycerol (TAG). When compared with Δ-s-tether cells, reductions in normalized phospholipid levels are even more pronounced in *sac1*^*ts*^ Δ-s-tether cells incubated at 37 °C for 1 h, which exhibit further decreases in PS, PE, and CDP-DAG and greater levels of DAG and TAG (Fig. 3*A*). Although most phospholipids are unaffected in *sac1*Δ and *sac1*^ts^ *inp52*Δ *inp53*Δ cells, PS levels are much reduced, albeit to a lesser degree than in *sac1*^ts^ Δ-s-tether cells. In fact, the combined phospholipid defects of *sac1*^ts^ and Δ-s-tether mutations is most evident in the reduction of CDP-DAG and PS levels.

Levels of some single-carbon chain lysophospholipids accumulate in strains lacking ER-PM tethers or the PI phosphatases (Fig. 3*B*). In Δ-s-tether cells, lysoPC (LPC) levels are comparable with WT levels, whereas lysoPE (LPE) levels are moderately elevated. In *sac1*Δ cells, LPC levels are equivalent to WT and substantially decreased in LPE. In contrast, the normalized levels of lysophospholipids in *sac1*^ts^ *inp52*Δ *inp53*Δ cells are ∼1.2- to 1.6-fold higher after incubation at 37 °C for 1 h. At 37 °C for 1 h, the inactivation of *sac1*^ts^ in Δ-s-tether cells increases the normalized level of LPC and results in a moderate increase in LPE levels. However, the inactivation of PI phosphatase activity in *sac1*^ts^ *inp52*Δ *inp53*Δ cells has the greatest effect on lysophospholipids and causes considerable LPC and LPE increases.

Because ER-PM MCSs impact sphingolipid biosynthesis (5), levels of ceramide and the complex sphingolipid IPC were analyzed in PI phosphatase and Δ-tether mutants (sphingolipid IPC derivatives, namely, mannose-inositol phosphorylceramide and mannose (inositol-P)2-phosphorylceramide, could not be definitively analyzed). As previously reported, the normalized lipidomic profile of Δ-s-tether cells shows increased ceramide levels and reduced amounts of IPC (Fig. 3*C*) (5). After 1-h incubation at 37 °C, *sac1*^ts^ inactivation in Δ-s-tether cells results in comparable changes (Fig. 3*C*). Inactivation of the PI phosphatases in *sac1*^*ts*^ *inp52*Δ *inp53*Δ cells also results in similar ceramide accumulation and lower levels of IPC. Under the conditions used in this study, the deletion of *SAC1* by itself reduces the normalized level of IPC. However, unlike previous reports (57), a concomitant accumulation of ceramide is not observed (although *SAC1* inactivation in tandem with *inp52*Δ *inp53*Δ clearly results in ceramide accumulation). Nevertheless, the disruption of sphingolipid biosynthesis in PI phosphatase and ER-PM tethers mutants suggests that they both participate in regulating ceramide incorporation into complex sphingolipids.

To compare rates of synthesis of phospholipids, logarithmic phased cells were cultured in synthetic medium and lipid synthesis was assayed following ^32^P pulse labeling. After preincubation at 30 or 37 °C for 1 h, [^32^P]H_3_PO_4_ was added to cultures for 2, 5, 10, and 20 min. Following lipid extraction and separation by thin-layer chromatography, the synthesis of PA, PS, PE, and PI/PC was measured in WT, *sac1*Δ, Δ-s-tether, *sac1*^ts^ Δ-s-tether, and *sac1*^ts^ *inp52*Δ *inp53*Δ cells (Fig. 3*D*). The deletion of *SAC1* has comparatively little impact on PA and PI/PC synthesis (though PS and PE synthesis is reduced), whereas phospholipid synthesis is markedly reduced in Δ-s-tether cells. At 37 °C for 1 h, which greatly increases phospholipid synthesis in WT cells, in *sac1*^ts^ *inp52*Δ *inp53*Δ cells phospholipid flux is severely inhibited (Fig. 3*D*). Thus, phospholipid synthesis is dependent on essential combined activities of *SAC1*, *INP52*, and *INP53*. In both Δ-s-tether cells at 30 °C and *sac1*^ts^ Δ-s-tether cells at 37 °C, the incorporation of ^32^P into PA and all other phospholipids assayed is almost blocked when compared with WT (Fig. 3*D*). Indeed, in *sac1*^ts^ Δ-s-tether cells the synthesis of PS and PE is barely detectable even after 20 min. When compared with their WT controls at the same temperature, phospholipid synthetic flux in *sac1*^ts^ Δ-s-tether cells is even further reduced than that in Δ-s-tether cells. These results directly show that phospholipid flux is dependent on the combined functional interaction of ER-PM MCS tethers and Sac1p.

### Genes involved in phospholipid biosynthesis suppress *sac1*^*ts*^ Δ-s-tether synthetic lethality

**Figure 4:**
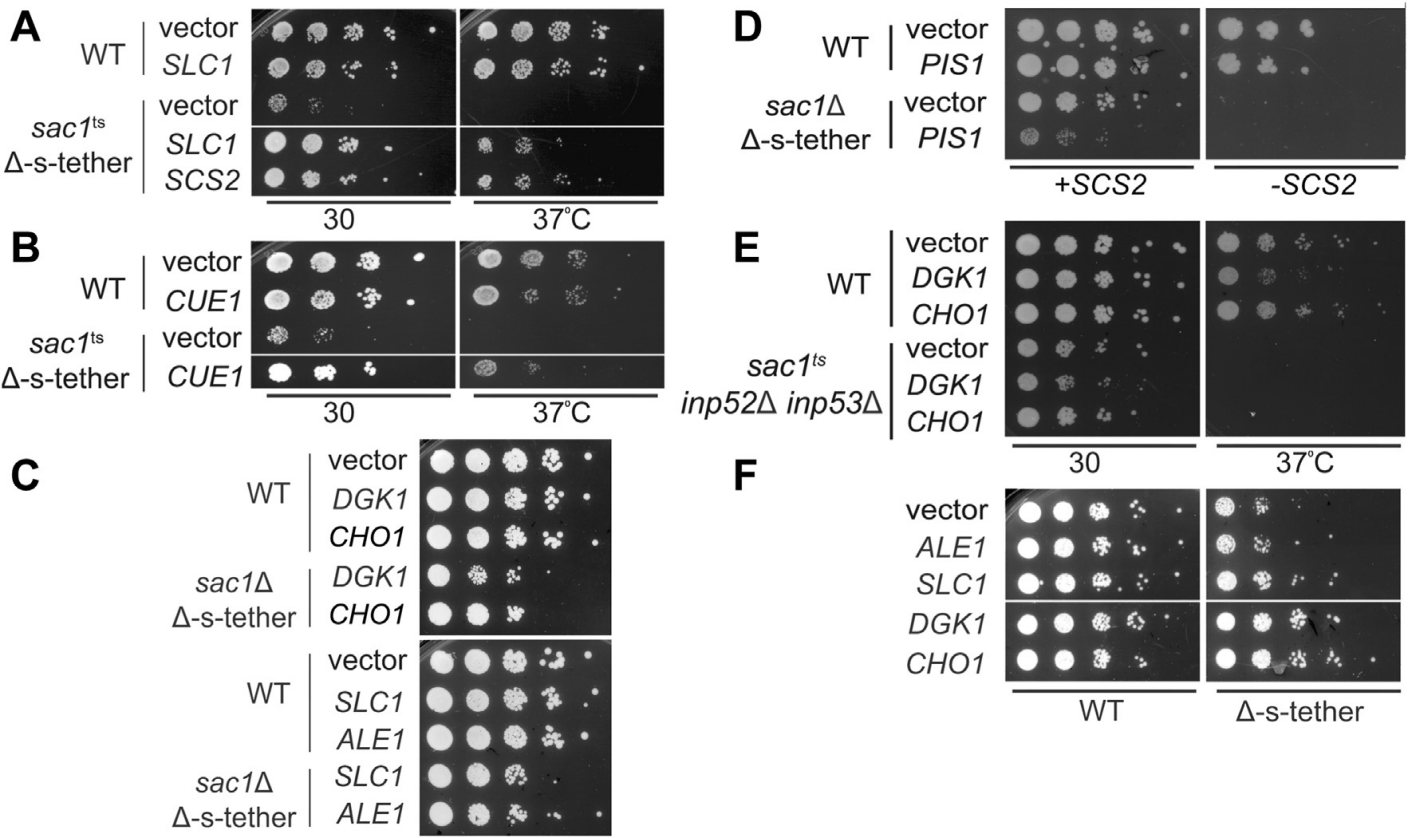
**Lipid biosynthesis genes and *CUE1* are dosage suppressors of the lethal inactivation of *SAC1* in Δ-s-tether cells.***A*, tenfold serial dilutions of WT (SEY6210) and *sac1*^ts^ Δ-s-tether (CBY6345) cells transformed with high-copy plasmids containing *SCS2* (pSCS2), a genomic fragment containing *SLC1* (pCB1350), and the vector control (YEplac195) cultured on selective solid medium for 3 to 5 days at the indicated temperatures. *B*, tenfold serial dilutions of WT and *sac1*^ts^ Δ-s-tether cells transformed with a high-copy plasmid containing a genomic *CUE1* fragment and the vector alone control cultured on selective solid medium for 3 to 5 days at the indicated temperatures. *C*, tenfold serial dilutions of WT and *sac1*Δ Δ-s-tether cells with high-copy plasmids containing *DGK1* (pCB1346), *CHO1* (pCB1352), *SLC1* (pCB1350), *ALE1* (pCB1382), or the corresponding vector control (YEplac181), grown and spotted onto solid synthetic medium at 30 °C for 3 days. Strains shown successfully grew after high-copy plasmid suppressors were transformed into WT and *sac1*Δ Δ-s-tether cells containing an *SCS2* plasmid (pSCS2), followed by *SCS2* counter-selection with the addition of 5′-fluoroorotic acid. *D*, tenfold serial dilutions of WT and *sac1*Δ Δ-s-tether cells containing episomal *SCS2* transformed with high-copy *PIS1* (pCB1345) in which the *SCS2* plasmid was either selected for (+*SCS2*) or counter-selected against (−*SCS2*), grown at 30 °C for 5 days. *E*, tenfold serial dilutions of WT (SEY6210) and *sac1*^ts^*inp52*Δ *inp53*Δ (AAY143) cells transformed with high-copy *DGK1*, *CHO1,* and the vector alone control. *F*, tenfold serial dilutions of WT and Δ-s-tether (CBY5838) cells containing high-copy *ALE1*, *SLC1*, *DGK1*, *CHO1,* and the vector alone control cultured at 30 °C for 4 days.

To identify genes that participate in the overlapping functions of *SAC1* and the ER-PM tether genes, a dosage suppressor selection was conducted to isolate extragenic suppressors of *sac1*^*ts*^ Δ-s-tether lethality. Following transformation with a high-copy (2 μ) plasmid library derived from Δ-s-tether cells (to avoid reisolation of the seven ER-PM tether genes), surviving *sac1*^*ts*^ Δ-s-tether transformants were selected after growth at 37 °C. Of the eight extragenic suppressors isolated, six corresponded to genomic fragments that included *SLC1,* which encodes an acyltransferase that converts lysophosphatidic acid to PA (Fig. 1*A*) (18, 19). Because *SLC1* can rescue growth in either the *sac1*^*ts*^ Δ-s-tether conditional mutant or *sac1*Δ Δ-s-tether cells, *SLC1* represents a bypass suppressor that circumvents the combined essential functions of *SAC1* and ER-PM tethers (Fig. 4*A*). As with all bypass suppressors, the mechanism of *SLC1* suppression of *sac1*Δ Δ-s-tether lethality cannot involve reestablishing physical connections between Sac1p and the tether proteins; interactions between proteins cannot be restored if their respective genes are absent. The selection also twice identified *CUE1* as a weak allelic suppressor of *sac1*^*ts*^ Δ-s-tether cell lethality (Fig. 4*B*). Cue1p encodes an ER-localized ubiquitin-binding protein that recruits the ubiquitin-conjugating enzyme Ubc7p to the ER membrane for ER-associated degradation of misfolded proteins during ER stress (58, 59). (*UBC7* overexpression was insufficient to suppress *sac1*^*ts*^ Δ-s-tether growth defects.) Given that *SLC1* was a strong suppressor with a clear link to the phospholipid defects in PI phosphate phosphatases and Δ-s-tether mutants, we focused exclusively on the mode of *SLC1* suppression. Moreover, the low number of *CUE1* isolates (N = 2) suggests that the genetic selection was not saturated and other potential suppressors might still be identified.

If *SLC1* overexpression suppresses *sac1*Δ Δ-s-tether lethality by boosting phospholipid levels through increased PA biosynthesis, we hypothesized that other PA-synthesizing genes might also bypass *sac1*Δ Δ-s-tether lethality (Fig. 4*C*). Like *SLC1*, overexpression of either *ALE1* or *DGK1* on high-copy plasmids rescued *sac1*Δ Δ-s-tether cell growth (Fig. 4*C*). *ALE1* and *DGK1* encode lysophospholipid acyltransferase and diacylglycerol kinase, respectively, both of which lead to PA biosynthesis. The enzymatic activity of the phosphatidate phosphatase Pah1p acts in opposition to Dgk1p by dephosphorylating PA to yield DAG (Fig. 1*A*) (60). We tested if *PAH1* deletion would phenocopy *DGK1* overexpression as a bypass suppressor of *sac1*Δ Δ-s-tether lethality. Unfortunately, after multiple attempts, viable *pah1*Δ *sac1*Δ Δ-s-tether transformants could not be isolated. Nonetheless, these results suggest that ER-PM MCSs and *SAC1* together affects the availability of PA as a precursor for the synthesis of CDP-DAG, which in turn serves as a substrate for PI and PS generation (Fig. 1*A*).

For *sac1*Δ Δ-s-tether suppression, we tested if PI or PS is the relevant phospholipid by overexpressing *PIS1* and *CHO1* (Fig. 4, *C* and *D*). *PIS1* encodes PI synthase, which produces the PI precursor for all phosphoinositide synthesis, whereas PS is produced by *CHO1*, which encodes PS synthase (61, 62)*.* Although *CHO1* is a strong bypass suppressor of *sac1*Δ Δ-s-tether lethality, *PIS1* overexpression failed to suppress the growth defect (Fig. 4, *C* and *D*). To further delineate the mode of suppression, we tested if high-copy *DGK1* or *CHO1* rescued PI phosphate phosphatase dysfunction in *sac1*^ts^ *inp52*Δ *inp53*Δ cells. At 37 °C, neither *DGK1* nor *CHO1* suppressed *sac1*^ts^ *inp52*Δ *inp53*Δ lethality, suggesting that these high-copy suppressors do not affect PI phosphate phosphatase activity *per se* (Fig. 4*E*). However, in the absence of choline the growth defect of Δ-s-tether cells is partially rescued by *DGK1* and *CHO1* (Fig. 4*F*). On the other hand, *ALE1* and *SLC1* overexpression has little effect on the growth of Δ-s-tether cells (Fig. 4*F*). Collectively these results suggest that the function of ER-PM contact sites, as relates to Sac1p activity, affects phospholipid flux from PA through the PS branch of the CDP-DAG biosynthesis pathway. The increased expression of these specific phospholipid biosynthetic genes rescues the overlapping lipid defects exacerbated by *SAC1* deletion in Δ-s-tether cells.

### Lipid biosynthetic genes rescue PM phospholipid defects in *sac1*^ts^ Δ-s-tether cells

**Figure 5:**
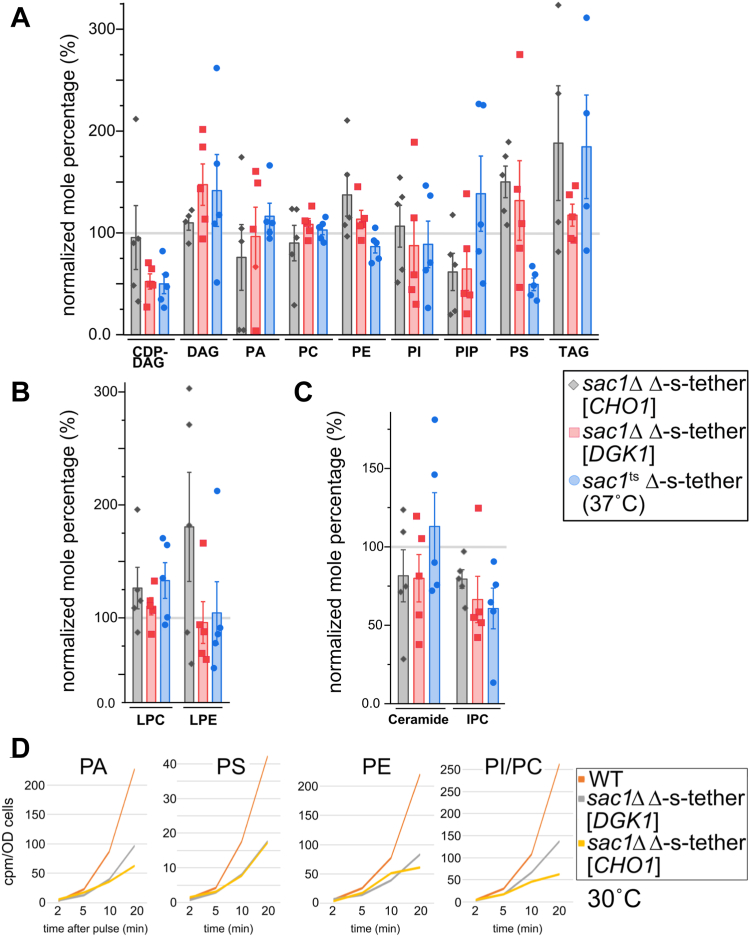
**Lipidomics analysis of high-copy suppressors of *sac1*Δ Δ-s-tether lethality.***A*, phospholipid composition of *sac1*^ts^ Δ-s-tether (CBY6345) cells incubated at 37 °C for 1 h, and *sac1*Δ Δ-s-tether cells containing high-copy *DGK1* (CBY6508) and high-copy *CHO1* (CBY6522) cultured at 30 °C, expressed as a normalized mole percentage relative to WT (set as 100%) cultured under the same conditions. *B*, lysophospholipid composition of the cells in (*A*), shown as a mole percentage relative to WT. *C*, ceramide and IPC composition of the cells in (*A*), as a normalized mole percentage relative to WT. The lipidomics data represent the mean ± SEM derived from five independent samples. *D*, pulse labeling analysis of phospholipid synthetic flux in *DGK1*- and *CHO1*-suppressed *sac1*Δ Δ-s-tether cells at 30 °C, as compared with WT. As per Figure 3*D*, glycerophospholipids were extracted 2, 5, 10, and 20 min after addition of ^32^P to log-phase cells, and lipids were separated *via* thin-layer chromatography and quantified. Each time point for each strain represents the average of duplicate independent analyses. CDP, cytidine diphosphate; DAG, diacylglycerol; IPC, inositol phosphoryl-ceramide; LPC, lysophosphatidylcholine; LPE, lysophosphatidylethanolamine; PA, phosphatidic acid; PC, phosphatidylcholine; PE, phosphatidylethanolamine; PI, phosphatidylinositol; PIP, phosphatidylinositol phosphate; PS, phosphatidylserine; TAG, triacylglycerol.

Given the altered lipid levels in *sac1*^ts^ Δ-s-tether cells (Fig. 3), we hypothesized that high-copy suppressors of *sac1*^ts^ Δ-s-tether lethality correct lipid metabolism defects in these cells. In fact, *DGK1* and *CHO1* dosage suppressors restored levels of most affected phospholipids near to WT levels (Fig. 5*A*). When compared with *sac1*^ts^ Δ-s-tether cells grown at 37 °C for 1 h, the normalized lipidomic profiles of *sac1*Δ Δ-s-tether cells rescued by high-copy *CHO1* or *DGK1* show increases in PS and PE levels, and *CHO1* and *DGK1* suppression confers reductions in DAG and TAG accumulation, respectively. Consistent with previous reports, high-copy *CHO1* generally increases both PE and PS levels, and *DGK1* overexpression conferred similar effects (27). Otherwise, *CHO1* and *DGK1* suppression in *sac1*Δ Δ-s-tether cells has variable effects on other phospholipids, where *CHO1* rescued the low level of CDP-DAG but *DGK1* did not. The results suggest that suppression of *sac1*Δ Δ-s-tether lethality correlates with increases in PE and PS levels and decreases in DAG or TAG.

The impact on lysophospholipids by *DGK1* and *CHO1* suppression of *sac1*Δ Δ-s-tether lethality is minor. *CHO1* overexpression in *sac1*Δ Δ-s-tether cells does not affect LPC, although LPE levels increased compared with *sac1*^ts^ Δ-s-tether cells at 37 °C for 1 h (Fig. 5*B*). *DGK1* overexpression elicits relatively modest if any changes. Given the lack of substantial changes in LPC and LPE levels between the suppressed *sac1*Δ Δ-s-tether strains and *sac1*^ts^ Δ-s-tether cells at 37 °C, the mechanism of *CHO1* and *DGK1* suppression is unlikely to involve lysophospholipid regulation.

As a component of the “SPOTS” (serine palmitoyltransferase, Orm1, Orm2, Tsc3, and Sac1) regulatory complex, *sac1*Δ affects the biosynthesis of sphingolipids from ceramide, as does the deletion of ER-PM tethers (Fig. 3*C*) (5, 63, 64). Tricalbins transfer ceramide from the ER to the Golgi for IPC synthesis (64), and the deletion of all tricalbin genes in Δ-s-tether cells likely impedes the normal sphingolipid synthetic process. As compared with *sac1*^ts^ Δ-s-tether cells at 37 °C for 1 h, *DGK1* or *CHO1* overexpression in *sac1*Δ Δ-s-tether cells shows reduced ceramide accumulations, which correlates with the minor increases in IPC levels observed (Fig. 5*C*). These results suggest that *DGK1* or *CHO1* overexpression partially restores sphingolipid biosynthesis in *sac1*Δ Δ-s-tether cells. Taken together, however, these results suggest that *DGK1* and *CHO1* overexpression rescues *sac1*Δ Δ-s-tether lethality by restoring normal phospholipid, DAG, and TAG levels, as opposed to suppressing sphingolipid synthesis defects.

Given that steady-state levels of phospholipids in *DGK1*- or *CHO1*-suppressed *sac1*Δ Δ-s-tether cells are generally returned to WT levels (Fig. 5*A*), a restoration of phospholipid synthetic flux might also be predicted. To test if phospholipid flux is rescued in suppressed *sac1*Δ Δ-s-tether cells, extracted lipids from logarithmic phased cells were assayed following ^32^P pulse labeling (Fig. 5*D*). As a proportion of the synthetic rate in WT cells under the same growth conditions, the synthesis of PA, PS, PE, and PI/PC increased in *DGK1*- or *CHO1*-suppressed *sac1*Δ Δ-s-tether cells compared with the lack of phospholipid synthesis in *sac1*^ts^ Δ-s-tether at 37 °C (Figs. 3*D* and 5*D*). Although phospholipid flux was not completely restored to WT levels in the suppressed strains, the moderate increases in synthetic rates account for the observed increases in steady-state phospholipid levels. Bypass suppressors of *sac1*Δ Δ-s-tether cells restore limited phospholipid flux irrespective of any physical interaction between Sac1p and ER-PM tethers.

### *CHO1* and *DGK1* suppression of *sac1*Δ Δ-s-tether lethality through phospholipid synthesis without restoring normal cER-PM association

**Figure 6:**
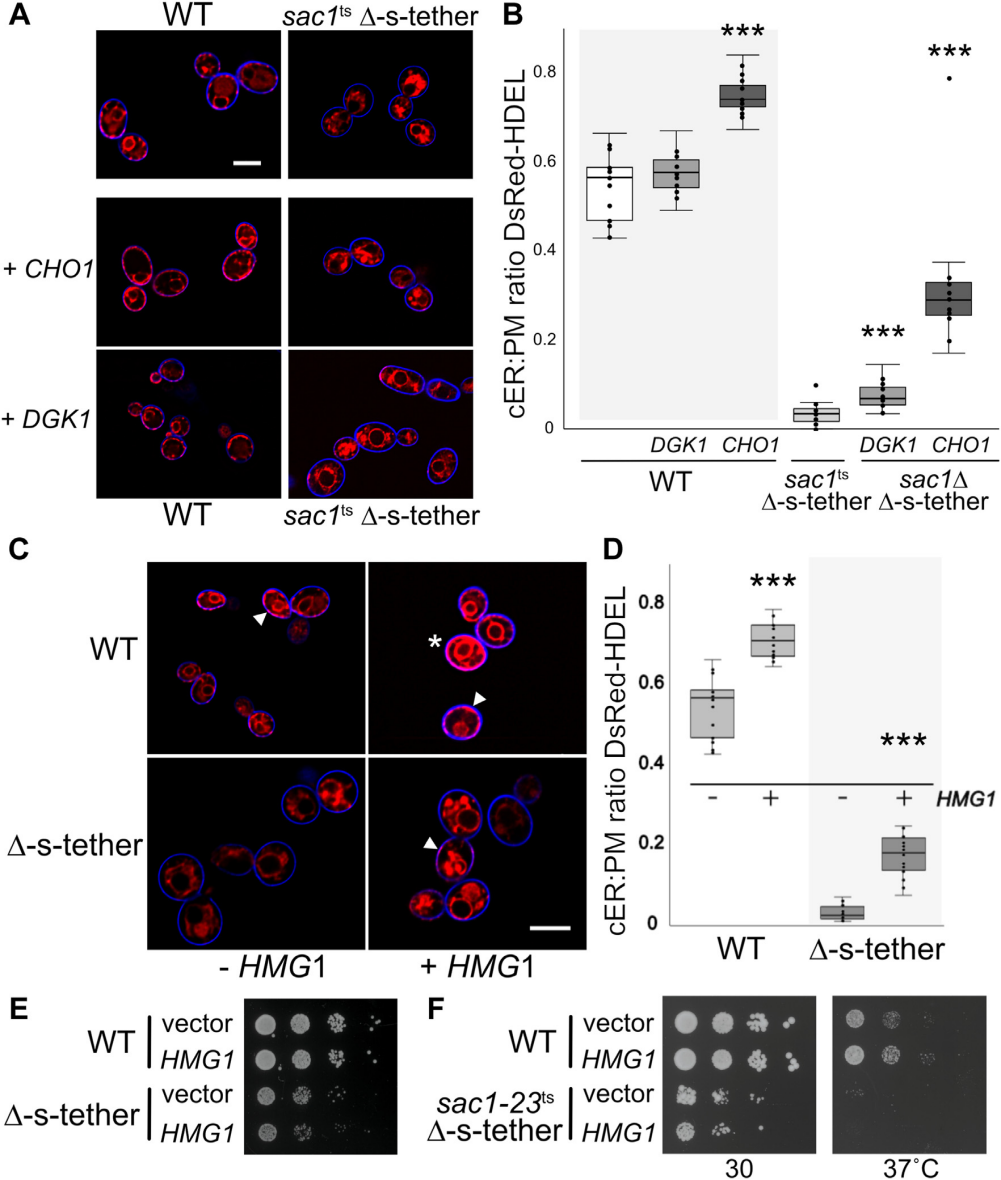
**High-copy *DGK1*, *CHO1*, or *HMG1* increase cER, but *HMG1* overexpression does not suppress *sac1***^**ts**^**Δ-s-tether lethality.***A*, representative images of endoplasmic reticulum–stained DsRed-HDEL in WT (SEY6210) and *sac1*^ts^ Δ-s-tether (CBY6345), or WT and *sac1*Δ Δ-s-tether cells expressing high-copy *DGK1* (CBY6508) or *CHO1* (CBY6522) and counterstained with a blue cell surface dye (MemBrite). Cells were incubated at 30 °C or at 37 °C for 1 h, as indicated. *B*, quantification of cell ratios of cER coverage per total distance of the PM perimeter, as corresponding to images shown in (*A*) (N = 20 per strain; ∗∗∗ *p* < 2 × 10^−5^). *C*, representative images of endoplasmic reticulum–marked DsRed-HDEL in WT and Δ-s-tether (CBY5838) cells expressing high-copy *HMG1* (+*HMG1*; pCB1402) or vector alone control (-*HMG1*; YEplac195) treated with the blue cortical dye and cultured at 30 °C. *D*, quantification of cell ratios of cER coverage per total distance of the PM perimeter corresponding to the images in (*C*) (N = 20 per strain; ∗∗∗*p* < 1 × 10^−9^). *E*, tenfold serial dilutions of WT and Δ-s-tether cells expressing high-copy *HMG1* or the vector control spotted on solid synthetic medium without supplemental 1 mM choline and cultured for 3 days at 30 °C. *F*, tenfold serial dilutions of WT and *sac1*^ts^ Δ-s-tether cells expressing high-copy *HMG1* or the vector control spotted on solid synthetic medium and cultured for 3 to 4 days at 30 °C or 37 °C. The scale bars represent 5 μm. cER, cortical endoplasmic reticulum; PM, plasma membrane.

To determine how the lipid biosynthetic suppressors affect ER-PM association, DsRed-HDEL was expressed in WT and *sac1*Δ Δ-s-tether cells transformed with high-copy *CHO1* or *DGK1* plasmids. Consistent with previous reports, *DGK1* overexpression in WT cells leads to increased DsRed-HDEL fluorescence in cytoplasmic ER, although without significant changes in cER at the PM (Fig. 6) (50, 64, 65). *DGK1* overexpression in *sac1*Δ Δ-s-tether cells causes a slight but significant increase in cER-PM association (Fig. 6, *A* and *B*). In contrast, high-copy *CHO1* in both WT and *sac1*Δ Δ-s-tether cells significantly increases ER-PM association relative to the vector alone controls (Fig. 6).

As formal possibilities, *sac1*Δ Δ-s-tether lethality might be suppressed by (*i*) reestablishing novel cER tethering with the PM; (*ii*) restoring lipid homeostasis between the ER and PM; (*iii*) indirectly promoting the cytoplasmic ER proliferation out to the cortex, thereby stochastically increasing ER-PM interaction. With respect to the latter possibility, *CHO1* overexpression results in the formation of unusually thick stacks of cytoplasmic ER reaching out toward the cell cortex that are reminiscent of karmellae, which represent an elaboration and proliferation of cytoplasmic ER (66). Karmellae are induced upon overexpression of *HMG1*, which encodes the isoprenoid synthetic enzyme 3-hydoxy-3-methylglutaryl coenzyme A reductase (66). In both WT and Δ-s-tether cells, *HMG1* overexpression results in significant ER expansion as observed using the fluorescent DsRed-HDEL ER-marker (Fig. 6, *C* and *D*). In this regard, the ER proliferation caused by *CHO1* overexpression resembles *HMG1*-induced karmellae. However, induction of cytoplasmic ER by high-copy *HMG1* does not rescue Δ-s-tether cells growth defects, indicating that ER proliferation cannot suppress defects in ER-PM MCSs (Fig. 6*E*). Moreover, this *HMG1*-induced cytoplasmic ER expansion does not suppress *sac1*^ts^ Δ-s-tether cell lethality when *HMG1* is overexpressed in *sac1*^ts^ Δ-s-tether cells (Fig. 6*F*). The fact that cytoplasmic ER expansion does not suppress growth defects in cells lacking ER-PM MCSs indicates that random ER association at the PM cannot substitute for directed membrane tethering.

**Supporting Figure S1:**
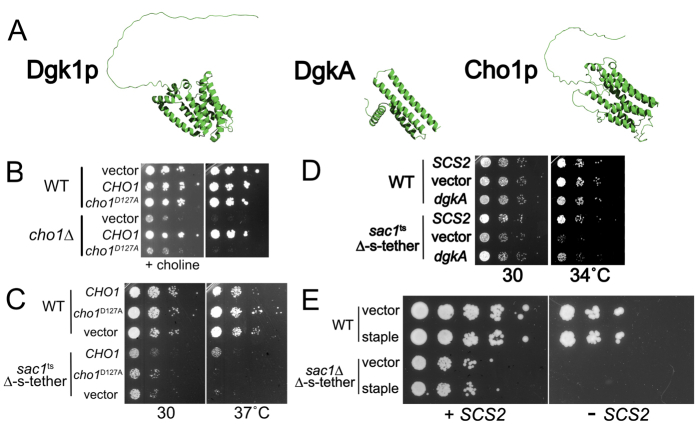
**Expression of an artificial ER-PM staple does not suppress *sac1*Δ Δ-s-tether lethality.***A*, monomeric protein structures of *S. cerevisiae* Cho1p and Dgk1p, and *E. coli* DgkA, as predicted by Alphafold 2 (118, 119). *B*, ten-fold serial dilutions of WT (SEY6210) and *cho1*Δ (CBY7553) cells transformed with high-copy plasmids bearing *CHO1* (pCB1351), the “catalytically-inactive” *cho1*^D127A^ (pCB1427), and the vector alone control (YEplac195), spotted onto solid synthetic media supplemented with (+ choline) or without 1 mM choline. Growth with choline is shown after 5 days and without choline for 3 days at 30˚C. *C*, ten-fold dilutions of WT and *sac1*^ts^ Δ-s-tether (CBY6345) cells transformed with a high-copy plasmids expressing *CHO1*, *cho1*^D127A^, or the vector control (YEplac195) incubated for 2.5 days at 30 or 37 °C as indicated. *D*, ten-fold dilutions of WT (SEY6210) and *sac1*^ts^ Δ-s-tether (CBY6345) cells transformed with a plasmid expressing *SCS2* (pSCS2), a vector control (pRS426), and a plasmid expressing bacterial *dgkA* from the P^*GAL1*^ yeast promoter (pCB1435) spotted on solid synthetic medium containing 4% galactose and 2% raffinose, incubated for 3 days at 30 and 34 °C. *E*, ten-fold serial dilutions of WT and *sac1*Δ Δ-s-tether cells containing episomal *SCS2* (CBY6146), transformed with the artificial staple plasmid (pCB1185) or the vector control (YCplac111), spotted onto solid synthetic media selecting for the *SCS2* plasmid (+*SCS2*), or counter-selecting against it (-*SCS2*) with the addition of 5’-FOA. The artificial staple is unable to suppress *sac1*Δ Δ-s-tether lethality in the absence of *SCS2*, a functionally important ER-PM tether.

Both Cho1p and Dgk1p have unstructured N-terminal extensions that might adopt the function of a membrane tether in the absence of ER-PM MCSs (Fig. S1*A*). To determine whether Cho1p has tethering capability or if its enzymatic activity is required, we tested the suppression *sac1*^ts^ Δ-s-tether growth defects by the conserved “catalytically dead” *CHO1*^D127A^ mutation (67). In the absence of choline in the growth medium, *CHO1*^D127A^ is nonfunctional and cannot complement the growth defect of *cho1*Δ cells (Fig. S1*B*). High-copy expression of *CHO1*^D127A^ cannot suppress *sac1*^ts^ Δ-s-tether lethality at 37 °C, indicating that Cho1p enzymatic activity is required to confer suppression. Unlike Cho1p or yeast Dgk1p, *Escherichia coli* diacylglycerol kinase (DgkA) lacks any conceivable tethering region but can enzymatically substitute for its yeast counterpart (Fig. S1*A*) (68, 69). Although the expression of DgkA in *sac1*^ts^ Δ-s-tether does not restore growth at 37 °C, bacterial DgkA does suppress *sac1*^ts^ Δ-s-tether grow defects at the semi-permissive growth condition of 34 °C (Fig. S1*D*). This result suggests that yeast Dgk1p does not physically attach membranes, but rather diacylglycerol enzymatic activity is necessary for suppression. Moreover, nonspecific restoration of ER-PM contact is inadequate to suppress *sac1*Δ Δ-s-tether lethality. ER-PM contact can be reestablished by an “artificial ER-PM staple,” which can ameliorate some growth defects of Δ-s-tether cells (5). When expressed in *sac1*Δ Δ-s-tether cells, however, the artificial staple is unable to rescue cell lethality (Fig. S1*E*). This result suggests that the functional interaction between Sac1p and native ER-PM tethers does not involve nonspecific cER physical attachment to the PM. Suppressors of *sac1*Δ Δ-s-tether lethality increase both cytoplasmic ER expansion and cER, but ER spread *en mass* is insufficient for suppression; suppression requires more than nonspecific reestablishment of ER and PM association. Instead, restoration of phospholipid metabolism bypasses the elimination of *SAC1* and ER-PM tethers.

### DAG, PS, and phosphoinositide localization is disrupted in cells lacking ER-PM tethers

**Figure 7:**
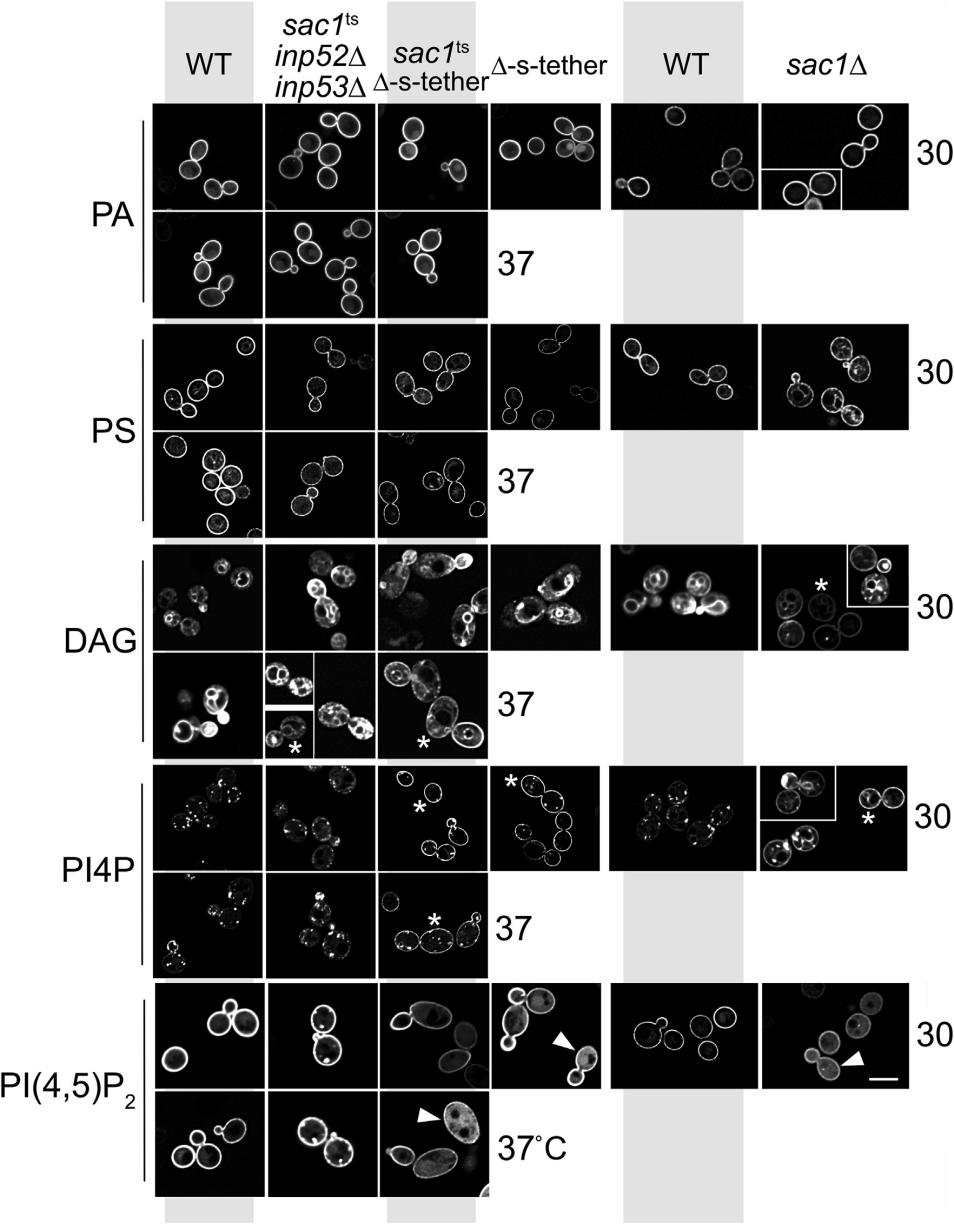
**Lipid distribution defects in phosphoinositide phosphatase and endoplasmic reticulum–plasma membrane tethering mutants.** Representative images of WT (SEY6210), *sac1*^ts^*inp52*Δ *inp53*Δ (AAY143), *sac1*^ts^ Δ-s-tether (CBY6345), and Δ-s-tether (CBY5838) cells expressing the PA-specific lipid probe GFP-Spo20^51–91^ (pRS426-G20), the PS-specific probe Lact-C2-GFP (Lact-C2-GFP-p416), the DAG-specific probe C1δ-GFP (pGPD416-C1δ-GFP), the PI4P-specific probe P4M-SidM-GFP (pCB1167), or the PI(4,5)P_2_ probe (pRS426GFP-2xPH(PLCδ)). *Asterisks* indicate examples of nonpolarized lipid distributions around the plasma membrane; *arrowheads* indicate examples of increased cytoplasmic PI(4,5)P_2_/pRS426GFP-2xPH(PLCδ) fluorescence. Log-phase cells were cultured at 30 °C, or incubated at 37 °C for 1 h, as indicated. For comparison, *sac1*Δ (CBY2809) cells and its congenic WT strain (BY4741) were transformed with the plasmids expressing each of the lipid probes and cultured at 30 °C before viewing. The scale bar represents 5 μm. DAG, diacylglycerol; PA, phosphatidic acid; PI(4,5)P_2_, phosphatidylinositol-4,5-bisphosphate; PI4P, phosphatidylinositol-4-phosphate; PS, phosphatidylserine.

**Figure 8:**
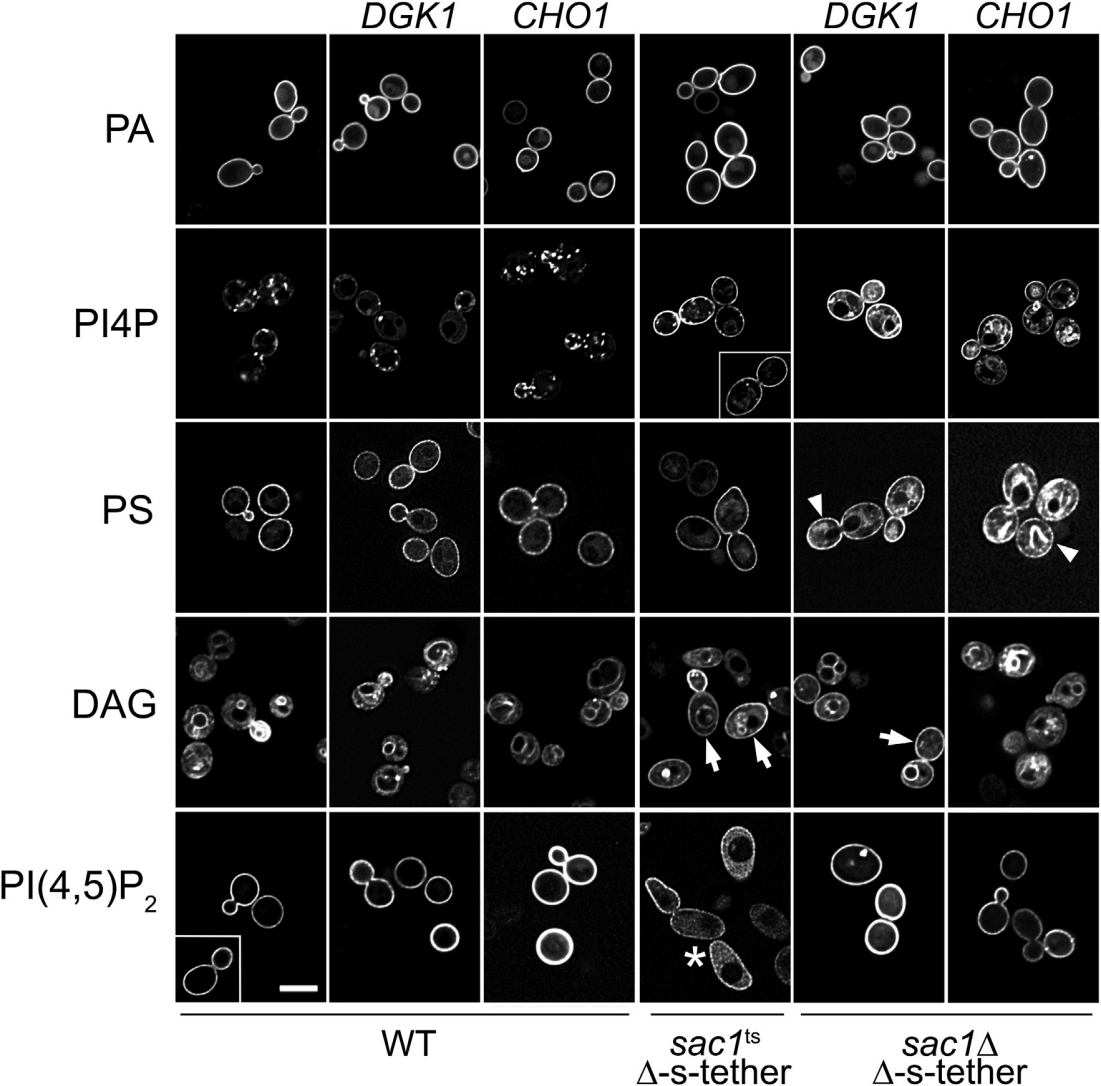
**Lipid distributions in *sac1*Δ Δ-s-tether cells expressing high-copy suppressors.** Representative images of WT (SEY6210), *sac1*^ts^ Δ-s-tether (CBY6345), and *sac1*Δ Δ-s-tether cells containing high-copy *DGK1* (pCB1346; CBY6508) and high-copy *CHO1* (pCB1352; CBY6522). Cells expressed PA (pRS426-G20), PS (Lact-C2-GFP-p416), DAG (pGPD416-C1δ-GFP), PI4P (pCB1167), or PI(4,5)P_2_ (pRS426GFP-2xPH(PLCδ)) fluorescent lipid probes. The *asterisk* indicates an example of increased cytoplasmic PI(4,5)P_2_/pRS426GFP-2xPH(PLCδ) fluorescence; arrows indicate examples of nonpolarized DAG distribution around the plasma membrane; *arrowheads* indicate increased PI4P/P4M-SidM-GFP fluorescence within internal membranes. Log-phase cells were imaged after incubation at 30 °C, and *sac1*^ts^ Δ-s-tether cells were visualized after a 1-h incubation at 37 °C. The scale bar represents 5 μm. DAG, diacylglycerol; PA, phosphatidic acid; PI(4,5)P_2_, phosphatidylinositol-4,5-bisphosphate; PI4P, phosphatidylinositol-4-phosphate; PS, phosphatidylserine.

The distribution of lipids within cellular membranes impacts their levels and metabolism (70). Given the role of MCSs in regulating lipid exchange between cER and the PM, we investigated if the defects in lipid composition revealed by lipidomic analyses are coupled with phospholipid mislocalization. Using specific fluorescent probes, the membrane localizations of DAG, PA, PS, PI4P, and PI(4,5)P_2_ were assessed by confocal microscopy in WT and *sac1*Δ cells as well as in *sac1*Δ Δ-s-tether cells containing suppressor plasmids, as compared with *sac1*^ts^ *inp52*Δ *inp53*Δ and *sac1*^ts^ Δ-s-tether cells after 1-h incubation at 37 °C (Figs. 7 and 8).

**Supporting Figure S2:**
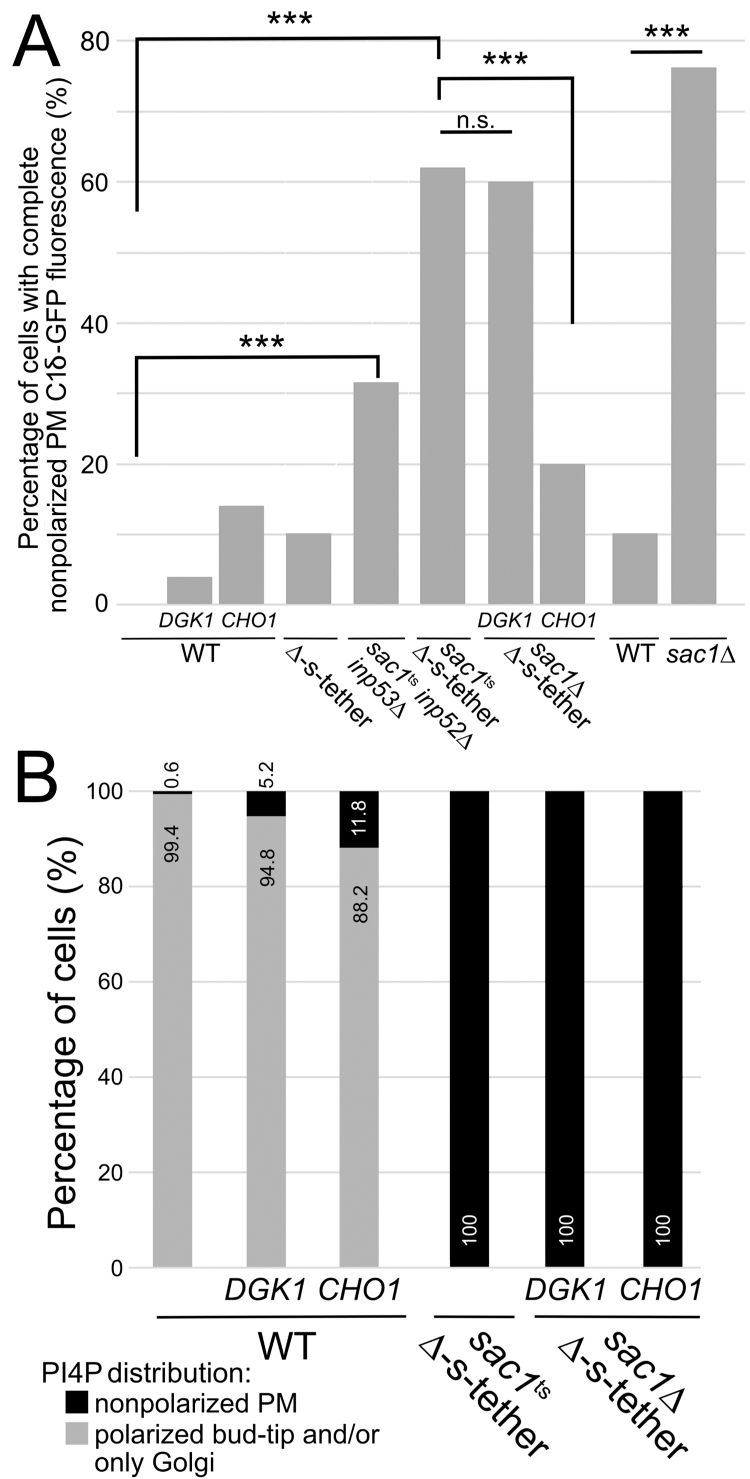
**Quantification of nonpolarized DAG/C1δ-GFP distribution at the PM, and PI4P/GFP-Spo20**^**51–91**^**distribution in PI phosphate phosphatase and ER-PM tether mutants.***A*, corresponding to the images in Figures 7 and 8, quantification of complete nonpolarized PM localization of the DAG-specific probe C1δ-GFP (pGPD416-C1δ-GFP) in WT (SEY6210) and Δ-s-tether (CBY5838) containing plasmids overexpressing *DGK1* (pCB1346), *CHO1* (pCB1352), or the vector control (YEplac181) at 30 °C. At 37 °C for 1 h, complete (nonpolarized) C1δ-GFP distribution along the PM is quantified in *sac1*^ts^*inp52*Δ *inp53*Δ (AAY143) and *sac1*^ts^ Δ-s-tether (CBY6345) cells (at 30 or 37 °C, WT cells only had polarized C1δ-GFP fluorescence at the PM). C1δ-GFP distribution at the PM is quantified in *sac1*Δ (CBY2809) cells relative to the congenic WT (BY4741) control. N = 50 cells for each strain; ∗∗∗*p* < 0.0001, n.s. = not statistically significant. *B*, quantification of the polarized PM distribution of the P^*ACT1*^-eGFP-P4M-SidM (pCB1167) PI4P-specific probe expressed in WT or *sac1*Δ Δ-s-tether cells overexpressing *DGK1* or *CHO1* as compared to vector alone cultured at 30 °C. *sac1*^ts^ Δ-s-tether cells were incubated at 37 °C for 1 h (at 30 or 37 °C, the polarized distribution of P^*ACT1*^-eGFP-P4M-SidM at the PM is equivalent).

Given the significant increases in DAG levels in Δ-s-tether and *sac1*^ts^ Δ-s-tether cells, we predicted that DAG distribution might be altered when observed with the DAG-specific lipid probe C1δ-GFP (70). In WT cells, C1δ-GFP fluorescence is primarily observed in the vacuolar membrane and polarized in the PM where it is restricted to budding daughter cells (Fig. 7) (71). However, in *sac1*Δ at 30 °C, and *sac1*^ts^ Δ-s-tether and *sac1*^ts^ *inp52*Δ *inp53*Δ at 37 °C for 1 h, C1δ-GFP is distributed all around the cell cortex indicating nonpolarized DAG localization in the mother and bud PM (Figs. 7 and S2*A*). In *sac1*Δ cells the PM defect in DAG polarization is most severe, which was a surprise given that the defect in *sac1*^ts^ *inp52*Δ *inp53*Δ cells (at 37 °C for 1 h or even at 30 °C, which were equivalent) was substantially less (Figs. 7 and S2*A*). Nonetheless, the changes in DAG levels and PM polarization in these mutant cells suggested further defects in the distribution of the phospholipids requiring DAG as their precursor.

Because PA biosynthetic genes (*SLC1*, *ALE1*, *DGK1*) suppress *sac1*^ts^ Δ-s-tether cell growth defects at 37 °C, we predicted changes in PA distribution in these and the other mutant cells. Consistent with previous reports, WT cells expressing the GFP-Spo20^51–91^ PA-specific lipid probe show fluorescence primarily along the PM with faint nuclear localization at 30 °C or 37 °C (Fig. 7) (69, 72). In *sac1*^ts^ Δ-s-tether or *sac1*^ts^ *inp52*Δ *inp53*Δ cells at 37 °C for 1 h, GFP-Spo20^51–91^ localization was indistinguishable from WT. Moreover, we observed no differences in GFP-Spo20^51–91^ distribution in *sac1*Δ or Δ-s-tether cells at 30 °C, as compared with congenic WT controls (Fig. 7). Despite the PM defects in DAG distribution in *sac1*^ts^ Δ-s-tether and other mutants, PA localization and steady-state levels appear unaffected.

To determine if the observed changes in DAG impacted phospholipid distribution other than PA, we analyzed the localization of phospholipids further along the CDP-DAG synthetic pathway (Fig. 1*A*). To observe PS distribution, the Lact-C2-GFP probe was visualized by confocal fluorescence microscopy in the various mutant cells (Fig. 7). In WT cells, PS is predominantly found in the PM with a polarized distribution somewhat concentrated at the cortex in small/medium buds (73). In many of the tether and PI phosphate phosphatase mutants, however, decreases in PS at the PM were detected. In Δ-s-tether cells, PS Lact-C2-GFP mean fluorescence is reduced at the PM to 47% of WT (N = 20 cells), and a faint fluorescence in internal membranes is detectable. Moreover, in *sac1*^ts^ Δ-s-tether and *sac1*^ts^ *inp52*Δ *inp53*Δ cells incubated at 37 °C for 1 h, the PS Lact-C2-GFP mean fluorescence at the PM is 42% and 40% compared with WT, respectively (N = 20 cells). The fluorescence was less affected in *sac1*Δ cells, in which PS Lact-C2-GFP was 70% of its congenic WT (N = 20 cells), although an elevation in internal membrane fluorescence is evident. Consistent with reductions in PS levels as shown by lipidomics (Fig. 3*A*), PS in Δ-s-tether, *sac1*^ts^ Δ-s-tether, and *sac1*^ts^ *inp52*Δ *inp53*Δ cells is significantly reduced in the PM.

Because ER/PM localization of PS and PI4P are interdependent due to a counter-exchange mechanism (74, 75), we analyzed *sac1*Δ, Δ-s-tether, *sac1*^ts^ Δ-s-tether, and *sac1*^ts^ *inp52*Δ *inp53*Δ cells to determine if the changes in PS levels are coupled with altered PI4P distribution. When expressed in WT cells, the P4M-SidM-GFP lipid probe shows PI4P localization in the Golgi and secretory vesicles and polarized in the PM sites only at the bud tip (76, 77) (Fig. 7). As previously reported, PI4P in *sac1*Δ and Δ-s-tether cells is not polarized within the PM, where P4M-SidM-GFP fluorescence spreads throughout the PM in both mother and daughter cells (5, 7). The same defect is observed in *sac1*^ts^ Δ-s-tether cells at 37 °C for 1 h, where PI4P is redistributed uniformly throughout the PM and fewer Golgi puncta are observed (Figs. 7 and S2*B*). In *sac1*^ts^ *inp52*Δ *inp53*Δ cells cultured at 30 or 37 °C for 1 h (N = 65), P4M-SidM-GFP fluorescence is uniformly concentrated along the PM in irregular puncta, which is suggestive of previous reports of deep phosphoinositide-enriched PM invaginations observed in PI phosphate phosphatase mutants (Fig. 7) (78, 79). The PM redistribution of PI4P in these mutant cells is consistent with the interdependence of PI4P and PS transfer between the ER and PM.

Because PI4P is a substrate for the synthesis of other phosphoinositides, namely, PI(4,5)P_2_ in the PM, we tested if PI(4,5)P_2_ distribution is altered by viewing the PI(4,5)P_2_-binding probe GFP-2xPH(PLCδ) when expressed in *sac1*Δ, Δ-s-tether, *sac1*^ts^ Δ-s-tether, and *sac1*^ts^ *inp52*Δ *inp53*Δ cells. Similar to WT cells, all mutants expressing the GFP-2xPH(PLCδ) probe show PI(4,5)P_2_ at the cell cortex around the PM. In 71% *sac1*^ts^ *inp52*Δ *inp53*Δ cells (N = 52), bright GFP-2xPH(PLCδ) puncta are also observed along the PM. In the other mutants, cells also exhibit increased cytoplasmic GFP-2xPH(PLCδ) fluorescence (Fig. 7). These results suggest that phospholipid and PI4P defects in PI phosphate phosphatase and ER-PM tether mutants lead to changes in PI(4,5)P_2_ distribution.

### Suppressors of *sac1*Δ Δ-s-tether lethality partially restore intracellular PI4P and PI(4,5)P_2_ distribution and cause PS accumulation in intracellular membranes

If *sac1*Δ Δ-s-tether lethality is due to defects in lipid metabolism, then rescue by high-copy bypass suppressors is predicted to restore at least some aspect of phospholipid distribution and synthesis. When expressed in WT cells, high-copy *DGK1* or *CHO1* does not appreciably affect the localization of lipid probes detecting PA, PI4P, or PS distribution (Fig. 8). Normal PA distribution is also unaffected in *sac1*Δ Δ-s-tether cells rescued by either the *DGK1* or *CHO1* suppressors. PI4P and PS distributions are, however, markedly different in the suppressed *sac1*Δ Δ-s-tether cells (Fig. 8). In WT or *sac1*^ts^ Δ-s-tether cells incubated at 37 °C for 1 h, PS localization is primarily detected at the cell cortex as shown by the Lact-C2-GFP probe, although, as previously described, PM Lact-C2-GFP fluorescence is considerably less when expressed in *sac1*^ts^ Δ-s-tether cells (Figs. 7 and 8). In all *sac1*Δ Δ-s-tether cells, whether suppressed by high-copy *DGK1* or *CHO1*, Lact-C2-GFP cortical fluorescence is nearly restored to WT levels (86% and 91% in *DGK1-* and *CHO1-*suppressed cells, respectively, relative to WT) and fluorescence is also evident in internal membranes. The observed increase in PS in intracellular membranes is consistent with PS accumulation in cytoplasmic ER that overproliferates in these cells (Figs. 5*A* and 8). Because of the dependency of PI4P localization on PS, we predicted that high-copy *DGK1* or *CHO1* suppression of *sac1*Δ Δ-s-tether growth defects would affect PI4P distribution between the ER and PM. As shown above (Figs. 7 and 8), P4M-SidM-GFP fluorescence in WT cells indicates PI4P at bud tips and the Golgi, whereas the PI4P distribution in *sac1*^ts^ Δ-s-tether cells is uniformly spread along the PM. Although this nonpolarized PI4P distribution is still observed in *sac1*Δ Δ-s-tether cells overexpressing *DGK1* or *CHO1* (albeit with less intensity, particularly in *sac1*Δ Δ-s-tether cells overexpressing *CHO1*), all these cells exhibit intense PI4P fluorescence in nonpunctate internal membranes consistent with cytoplasmic ER (Fig. 8). These changes in PI4P distribution correlate with rescue of PI(4,5)P2 defects as shown by GFP-2xPH(PLCδ) fluorescence. The general cytoplasmic GFP-2xPH(PLCδ) fluorescence observed in *sac1*^ts^ Δ-s-tether cells at 37 °C for 1 h is absent in all *sac1*Δ Δ-s-tether cells overexpressing *DGK1* or *CHO1*. These suppressors completely restore PI(4,5)P_2_ distribution as observed in WT cells (Fig. 8).

Although high-copy *CHO1* was effective in returning DAG distribution and levels closer to WT, *DGK1* suppression did not have a similar effect (Figs. 5*A*, 8, and S2*A*). As shown by C1δ-GFP fluorescence, uniform DAG localization in *CHO1-*suppressed *sac1*Δ Δ-s-tether cells is partially rescued, whereas *DGK1* suppression exhibits nonpolarized DAG distribution nearly identical to *sac1*^ts^ Δ-s-tether cells incubated at 37 °C for 1 h (Figs. 8 and S2*A*). We conclude that defects in DAG distribution are not a primary cause of lethality between *sac1*Δ and Δ-s-tether mutations because DAG polarization at the PM is not rescued by high-copy *DGK1* and only partially suppressed by *CHO1* overexpression in *sac1*Δ Δ-s-tether cells.

### Increased expression of *OSH6* suppresses *sac1*^ts^ Δ-s-tether growth defects

**Figure 9:**
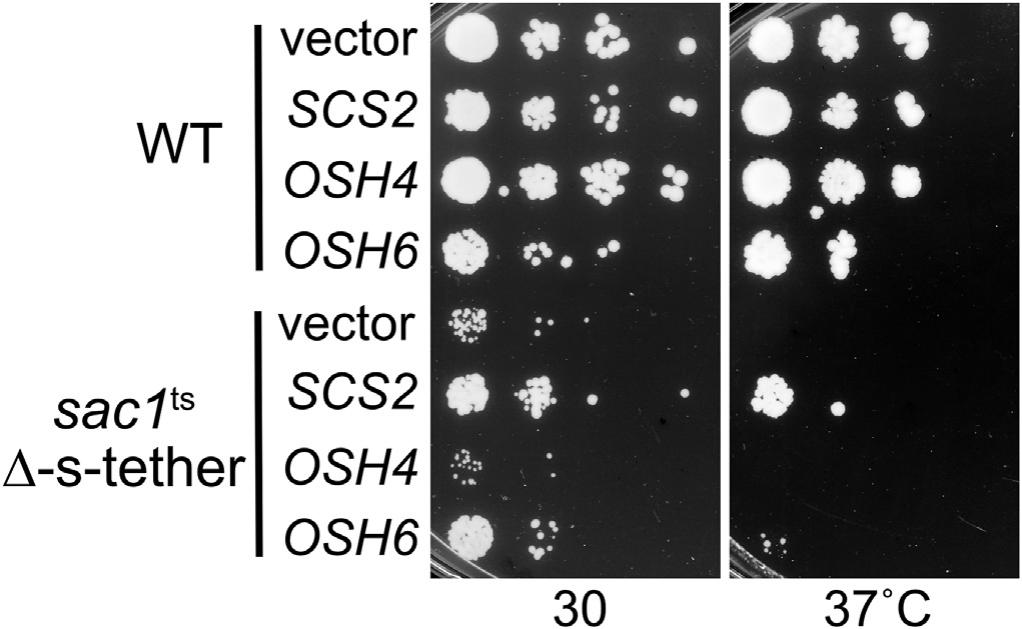
**High-copy expression of *OSH6* partially suppresses *sac1***^**ts**^**Δ-s-tether growth defects.** Tenfold serial dilutions of WT (SEY6210) and *sac1*^ts^ Δ-s-tether (CBY6345) cells containing high-copy *OSH4* (pCB241), *OSH6* (pCB237) plasmids, a vector control (pRS426), or *SCS2* (pSCS2) cultured at 30 °C for 4 days or 37 °C for 7 days.

Given that PS and PI4P are the most affected of the lipids tested in *sac1*^ts^ Δ-s-tether cells, we tested if the PS/PI4P lipid transfer protein Osh6p is an effector of the combined function of *SAC1* and ER-PM MCSs. As a member of the oxysterol-binding protein (OSBP)-related protein (ORP) family of soluble lipid transfer proteins, Osh6p represents one of seven yeast ORPs encoded by the *OSH1*-*OSH7* (OSBP homologue) genes (80, 81). Osh6p mediates PI4P/PS counter-directional transport in which Osh6p first transfers PS to the PM. At the PM, Osh6p reciprocally exchanges bound PS for PI4P and then returns to the ER with PI4P. The transport cycle is completed when PI4P is exchanged for PS in the ER (74, 75). Both Osh6p and Osh7p interact with the ER-PM tether Ist2p to facilitate PI4P/PS transfer between the PM and ER at MCSs (82, 83). We hypothesized that the lethality of *SAC1* deletion in Δ-s-tether cells might reflect a perturbation of the Osh6p-dependent cycle of PI4P/PS exchange at ER-PM MCSs. If so, Osh6p overexpression might rescue *sac1*^ts^ Δ-s-tether growth defects by boosting PI4P/PS counter-directional transport. As shown in Figure 9, a high-copy *OSH6* plasmid improves *sac1*^ts^ Δ-s-tether growth defects at 30 °C and suppresses *sac1*^ts^ Δ-s-tether lethality at 37 °C, although growth is poor. High-copy *OSH6* was not a bypass suppressor as it could not suppress the complete deletion of *SAC1* in Δ-s-tether cells. Osh4p, in contrast to Osh6p, is also a soluble lipid transfer protein but has an affinity for PI4P and sterols, but not PS (84). Unlike *OSH6*, *OSH4* on a high-copy plasmid failed to improve *sac1*^ts^ Δ-s-tether cell growth at 30 or 37 °C (Fig. 9). Because Osh4p and Osh6p do not share the same lipid affinities, other than binding PI4P, the Osh6p mode of suppression of *sac1*^ts^ Δ-s-tether defects specifically involves PS synthesis and transfer. Osh6p intermembrane lipid transfer, however, is ultimately dependent on levels of PS synthesized in the ER, as well as PI4P generated in the PM by Stt4p and eliminated in the ER by Sac1p. These results are consistent with a model where greater Osh6p expression confers enhanced PS transfer to the PM that partially rescues the reduced PM levels of PS in *sac1*^ts^ Δ-s-tether cells.

### The genomic expression profile of *sac1*^*ts*^ Δ-s-tether cells indicates constitutive responses to ER membrane stress and induction of autophagy gene expression

**Figure 10:**
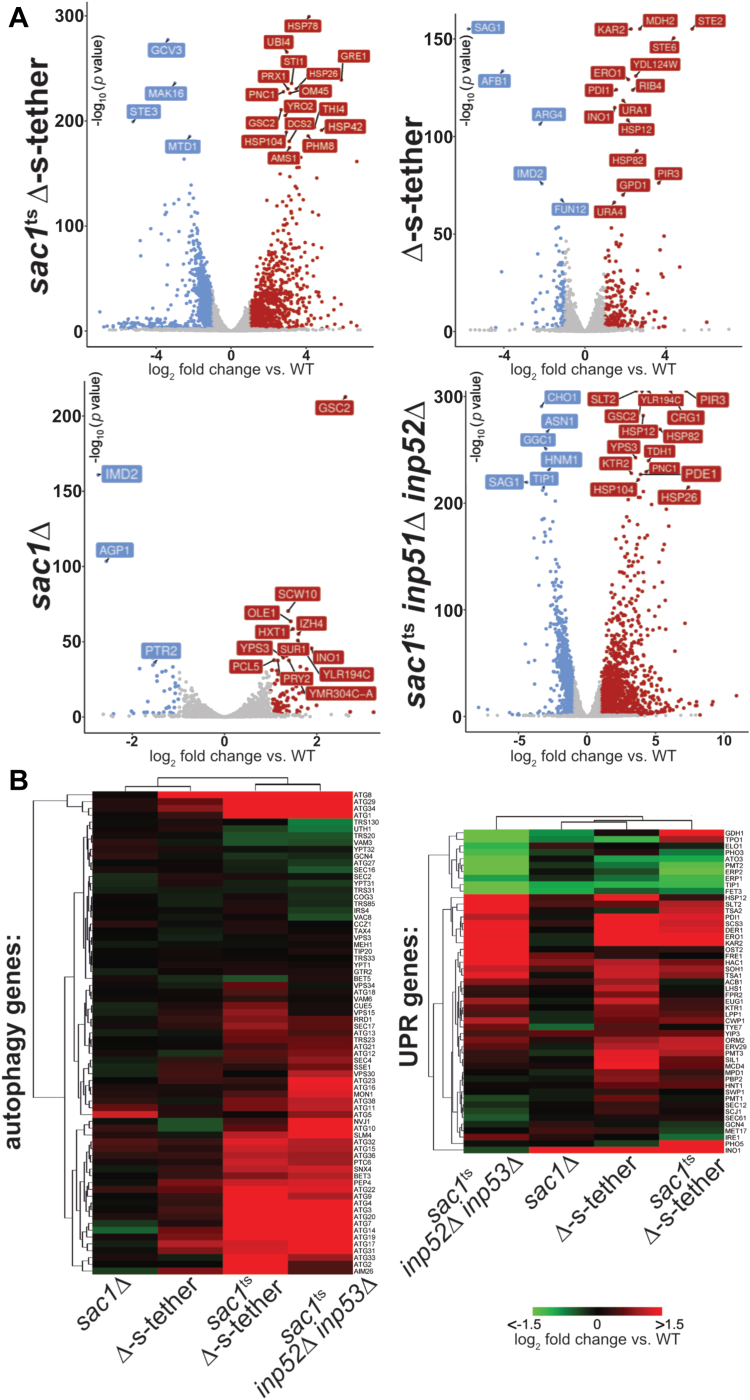
**Transcriptomic profiles of *sac1***^**ts**^**Δ-s-tether, Δ-s-tether, *sac1*Δ, and *sac1***^**ts**^***inp52*Δ *inp53*Δ.***A*, volcano plots showing relative transcript abundance in Δ-s-tether (CBY5898) at 30 °C, and *sac1*^ts^ Δ-s-tether (CBY6345) and *sac1*^ts^*inp52*Δ *inp53*Δ (AAY143) incubated at 37 °C for 1 h. Transcript changes are shown relative to WT (SEY6210) incubated under the same respective conditions. *sac1*Δ (CBY2809) was cultured at 30 °C relative to its congenic WT control (BY4741). Plots show the statistical significance of the difference in expression (negative log_10_-*p* value) *versus* log_2_-fold transcript changes, with examples of downregulated genes in *blue* and upregulated genes in *red*. *B*, heatmap analyses of autophagy and unfolded protein response (UPR) gene transcript changes relative to congenic WT controls for *sac1*^ts^ Δ-s-tether and *sac1*^ts^*inp52*Δ cells *inp53*Δ at 37 °C for 1 h, and for Δ-s-tether and *sac1*Δ cells at 30 °C. Autophagy genes were curated using the Saccharomyces Genome Database (SGD), and UPR genes were curated from Kimata *et al.* (120) and SGD. Upregulated genes for each strain are shown in *red*, and downregulated genes are indicated in *green*.

**Figure 11:**
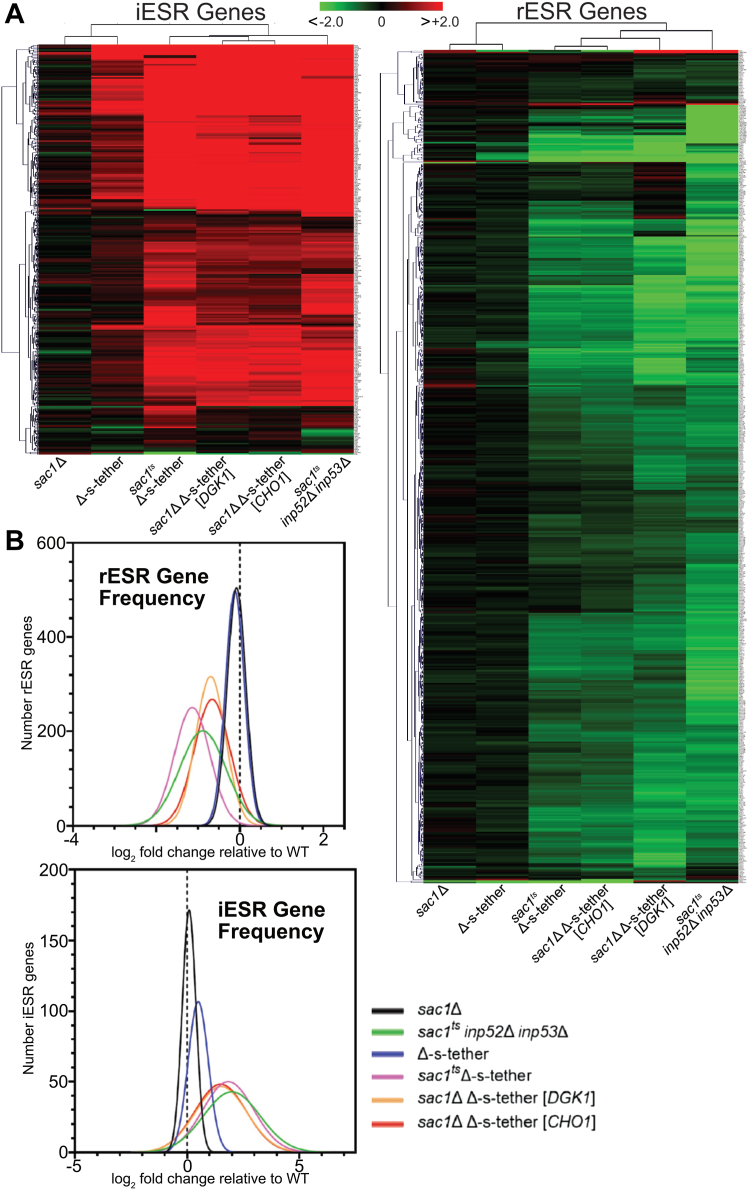
**Environmental stress response (ESR) transcript regulation in phosphoinositide phosphatase and endoplasmic reticulum–plasma membrane tethering mutants.** Heatmap analyses of *A*, iESR and rESR gene transcript changes for *sac1*Δ (CBY2809) and Δ-s-tether (CBY5898) cells at 30 °C, and *sac1*^ts^ Δ-s-tether (CBY6345) and *sac1*^ts^*inp52*Δ *inp53*Δ (AAY143) cells incubated at 37 °C for 1 h, relative to similarly cultured WT (SEY6210) cells. iESR and rESR transcript changes are also shown for *sac1*Δ Δ-s-tether cells rescued by either *DGK1* or *CHO1* overexpression. *sac1*Δ (CBY2809) cells cultured at 30 °C, relative to its congenic WT control (BY4741). *B*, graphical representations of the distribution of iESR and rESR gene transcript changes in *sac1*Δ, Δ-s-tether, and *sac1*^ts^ Δ-s-tether and *sac1*^ts^*inp52*Δ *inp53*Δ cells relative to their congenic WT controls at the same conditions.

**Supporting Figure S3:**
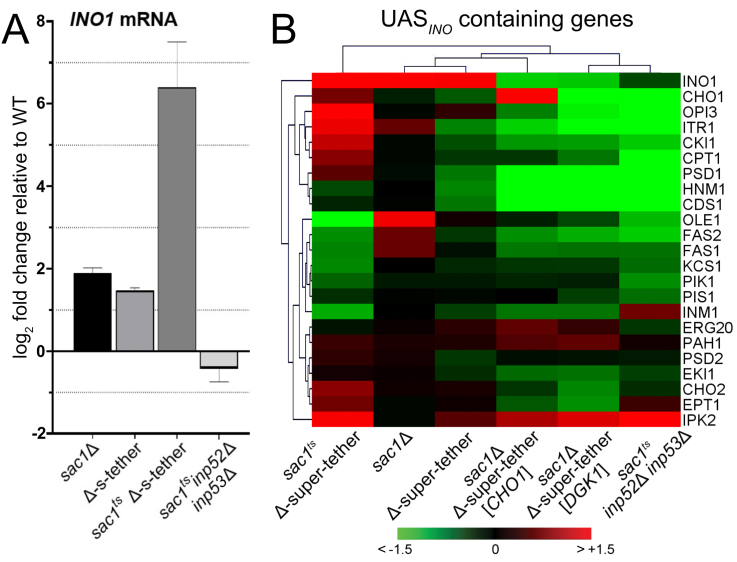
**Expression lipid biosynthetic genes under UAS**_***INO***_**transcriptional control in *sac1***^**ts**^**Δ-s-tether and *sac1***^**ts**^***inp52*Δ *inp53*Δ cells.***A*, relative to WT (SEY6210), quantification of *INO1* transcript levels in *sac1*^ts^*inp52*Δ *inp53*Δ (AAY143), *sac1*^ts^ Δ-s-tether (CBY6345) incubated for 1 h at 37 °C, and Δ-s-tether (CBY5838) at 30 °C. Relative to its congenic WT control (BY4741), *INO1* mRNA levels were also quantified in *sac1*Δ (BY4741) cells cultured at 30 °C. *B*, heat map analysis of transcriptional responses of UAS_*INO*_-regulated genes expressed in the strains described in panel *A*. Genes were curated as previously described (50). Downregulated genes are shown in *green*, and upregulated genes are shown in *red*.

**Supporting Figure S4:**
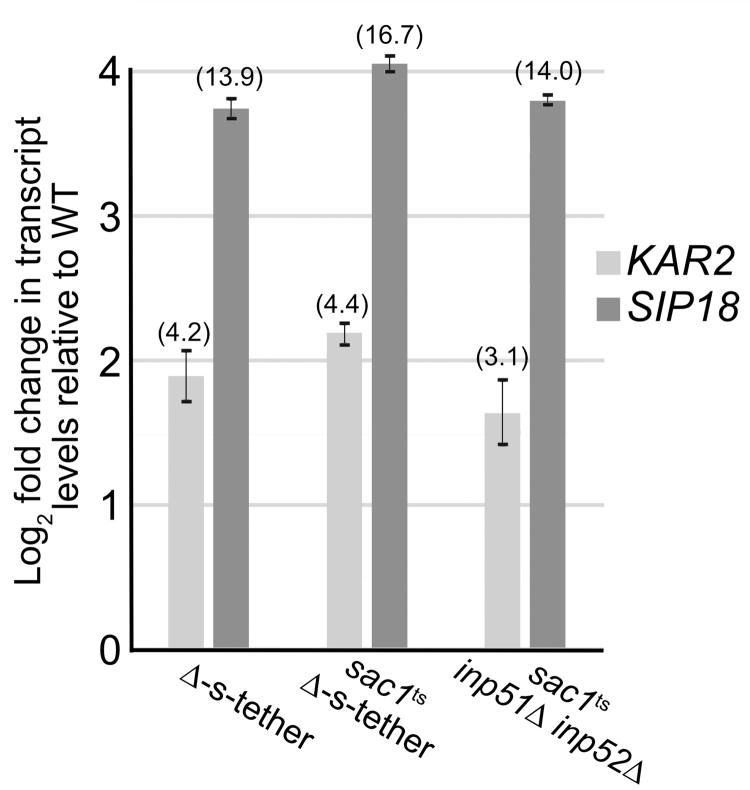
**qPCR validation of RNA-seq data.** Analyses of *KAR2* and *SIP18* transcript levels by RT-qPCR normalized to WT (SEY6210) levels for Δ-s-tether (CBY5838), *sac1*^ts^*inp52*Δ *inp53*Δ (AAY143) and *sac1*^ts^ Δ-s-tether (CBY6345) cells at 37 °C for 1 h. Absolute values of median fold changes are as shown; error bars indicate SEM. N = 3 independent cDNA samples.

We hypothesized that changes in global gene expression in *sac1*^ts^ Δ-s-tether cells might reveal the molecular nature of their growth defects. After incubation at 37 °C for 1 h, the transcriptome of *sac1*^ts^ Δ-s-tether cells was compared with WT using RNA deep sequencing analysis (RNA-seq) (Fig. 10*A*). Under these conditions, the transcription of 839 genes are downregulated in *sac1*^ts^ Δ-s-tether cells at least 2-fold (log_2_ ≤ 1) and 948 genes are upregulated 2-fold or more (log_2_ ≥ 1) compared with WT. (Significant changes are defined as those involving at least a 2-fold change in transcript levels relative to WT.) Specifically, ER-stress genes represented by unfolded-protein response (UPR)-induced genes (*e.g.*, *KAR2, DER1, PDI1*) are upregulated in *sac1*^ts^ Δ-s-tether cells, as are a subset of autophagy genes (*e.g.*, *ATG*1, *ATG8, ATG31*) (Fig. 10*B*). In addition, expression of many lipid biosynthetic genes under UAS_*INO*_ transcriptional control, including the key phospholipid synthetic gene *INO1* (encoding inositol-3-phosphate synthase), are significantly upregulated in *sac1*^ts^ Δ-s-tether cells (Fig. S3) (85). Taken together these changes in gene expression are integrated through the broader response elicited by the environmental stress response (ESR) pathway (Fig. 11). The ESR pathway is a global stress response pathway defined by two subsets of genes, which include those induced during general cellular stress (iESR) and a larger subset of transcriptionally repressed (rESR) genes, representing many associated with ribosomal proteins and ribosome biogenesis (86, 87). iESR genes are typically involved in protein catabolism, intracellular signaling, autophagy, and stress defense. Thus, defects in *sac1*^ts^ Δ-s-tether cells not only cause ER membrane stress but also elicit broader responses to general cellular stress as coordinated through ESR regulation. The validity of the RNA-seq results was confirmed by quantitative PCR analysis of the expression of *KAR2* and *SIP18*, performed in triplicate (Fig. S4).

We compared *sac1*Δ, Δ-s-tether and *sac1*^ts^ Δ-s-tether transcriptomic profiles to discern between transcript changes conferred by *SAC1* deletion *versus* the elimination of ER-PM tethers. To our surprise, the deletion of *SAC1* by itself imparts relatively minor effects relative to WT (Fig. 10*B*). The transcriptomic profile of *sac1*Δ cells reveals only 36 downregulated genes, whereas the expression of 76 genes increased. Overall, these changes mainly involve differences in metabolic gene expression (Fig. 10*A*). To provide a more comprehensive transcriptomic profile of yeast lacking phosphoinositide phosphate phosphatase activity, RNA-seq was conducted on *sac1*^ts^ *inp52*Δ *inp53*Δ cells incubated at 37 °C for 1 h. Although *sac1*Δ and *sac1*^ts^ *inp52*Δ *inp53*Δ cells share similar expression profiles, transcriptional responses to *sac1*^ts^ *inp52*Δ *inp53*Δ are far more extensive (Fig. 10*A*). Transcript levels of 892 genes are downregulated in *sac1*^ts^ *inp52*Δ *inp53*Δ cells and the expression of 1389 genes increase, when compared with WT levels. Repressed transcripts represented lipid biosynthesis (*CHO1, PSD1, OPI3*) and general metabolism pathways (*MTR4, RIX1*), while many upregulated transcripts represented autophagy (*e.g.*, *ATG1, ATG8, ATG31*), UPR (*e.g.*, *SLT2, KAR2, DER1, PDI1*), and osmotic stress pathway genes (*e.g.*, *GRE2, GRE3, SSA1*) (Fig. 10*A*). In contrast, the genome expression profile of Δ-s-tether cells revealed significant changes in gene expression affecting lipid biosynthesis genes and UPR regulators, as previously reported (Fig. 10) (50). Comparing the transcriptomic profiles of *sac1*^ts^ *inp52*Δ *inp53*Δ, Δ-s-tether, and *sac1*^ts^ Δ-s-tether cells indicates shared effects on ER stress and UPR activation (*e.g.*, induction of *KAR2, DER1, PDI1*), but differences in gene expression suggest synergistic amplification of responses when *SAC1* and tether gene disruptions are combined.

**Supporting Figure S5:**
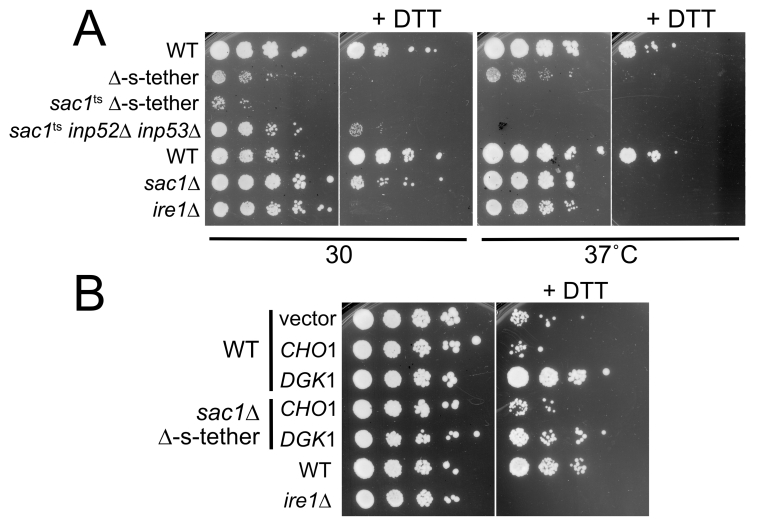
**Mutant sensitivities to ER stress.***A*, ten-fold dilutions of Δ-s-tether (CBY5838), *sac1*^ts^*inp52*Δ *inp53*Δ (AAY143), *sac1*^ts^ Δ-s-tether (CBY6345) cells matched with their congenic WT strain (SEY6210), and *ire1*Δ (CBY1048) and *sac1*Δ (CBY2809) controls with their congenic WT strain (BY4741). Strains were cultured on growth media with and without 3 mM DTT at 30 °C and 37 °C as indicated. *B*, ten-fold dilutions of WT (SEY6210) and Δ-s-tether (CBY5838) cells containing plasmids overexpressing *DGK1* (pCB1346), *CHO1* (pCB1352), or the vector control (YEplac181) on solid growth media with and without 3 mM DTT incubated at 30 °C.

As confirmation of the UPR induction revealed in the transcriptomic profiles, we tested if *sac1*Δ, *sac1*^ts^ *inp52*Δ *inp53*Δ, Δ-s-tether, and *sac1*^ts^ Δ-s-tether cells are sensitive to low doses of dithiothreitol (DTT) (Fig. S5*A*). In the presence of 3 mM DTT, growth is inhibited in mutant cells where the UPR pathway is dysfunctional (88, 89). For example, the deletion of *IRE1*, which encodes a key regulatory kinase for UPR activation, causes cell lethality in the presence of DTT (Fig. S5). Likewise, *sac1*Δ, *sac1*^ts^ *inp52*Δ *inp53*Δ, Δ-s-tether, and *sac1*^ts^ Δ-s-tether cells are also sensitive to 3 mM DTT (Fig. S5*A*). These results are consistent with the elevated ER stress in these mutants as determined by RNA-seq transcriptomics (Fig. 10*B*).

**Supporting Figure S6:**
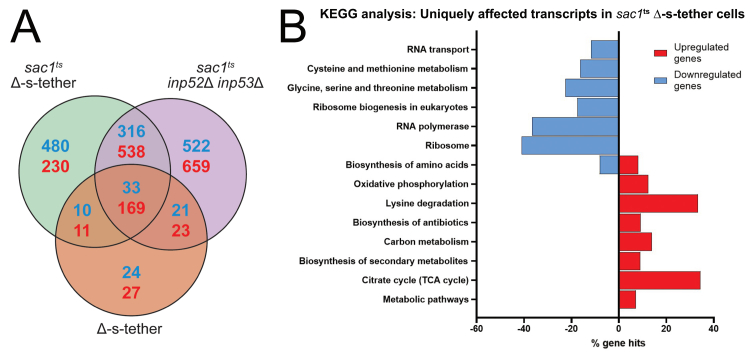
**Comparative analysis of gene expression in *sac1***^**ts**^**Δ-s-tether cells.***A*, Venn diagram indicating numbers of upregulated (*red*) and downregulated (*blue*) genes in *sac1*^ts^ Δ-s-tether (CBY6345) and *sac1*^ts^*inp52*Δ *inp53*Δ (AAY143) cells at 37 °C for 1 h, and Δ-s-tether (CBY5838) at 30 °C, relative to WT (SEY6210). *B*, KEGG categories of gene transcripts uniquely affected in *sac1*^ts^ Δ-s-tether cells relative to WT after incubation at 37 °C for 1 h.

**Supporting Figure S7:**
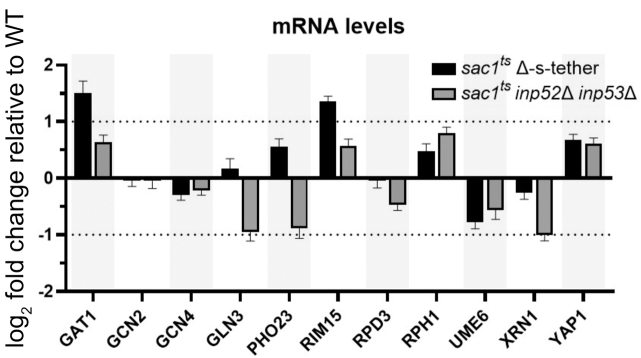
**Changes in expression of autophagy regulatory genes in *sac1***^**ts**^**Δ-s-tether and *sac1***^**ts**^***inp52*Δ *inp53*Δ cells.** RNA-seq analysis of transcript levels by autophagy activators (*GAT1*, *GCN2*, *GCN4*, *RIM15*, *RPH1*, *YAP1*) and autophagy repressors (*GLN3*, *PHO23*, *UME6*, *RPD3*, *XRN1*) in *sac1*^ts^ Δ-s-tether (CBY6345) and *sac1*^ts^*inp52*Δ *inp53*Δ (AAY143) cells relative to WT (SEY6210) at 37 °C for 1 h.

The *sac1*^ts^ Δ-s-tether transcriptomic profile shares 718 upregulated genes and 359 downregulated genes with the profiles of either *sac1*^ts^ *inp52*Δ *inp53*Δ cells or Δ-s-tether cells, or both (Fig. S6*A*). In *sac1*^ts^ Δ-s-tether and *sac1*^ts^ *inp52*Δ *inp53*Δ cells, but not Δ-s-tether cells, autophagy gene expression (*e.g.*, *ATG1*, *ATG8, ATG31*) is upregulated (Fig. 10*B*). These profile differences suggest that the regulation of autophagy responds to phosphoinositide phosphate phosphatase inactivation, and ER-PM tether deletions amplify the response. In these mutants, increased expression of autophagy transcriptional and translational activators (*GAT1*, *RIM15*, *RPD3*, *YAP1*) is observed and expression of several autophagy repressors (*UME6*, *XRN1*) is decreased (Fig. S7). The expression of most of these regulators is subject to Target of Rapamycin Complex 1 (TORC1) control, suggesting that *sac1*^ts^ Δ-s-tether and *sac1*^ts^ *inp52*Δ *inp53*Δ cells defects might affect TORC1-dependent responses to nutrient starvation (90).

**Supporting Figure S8:**
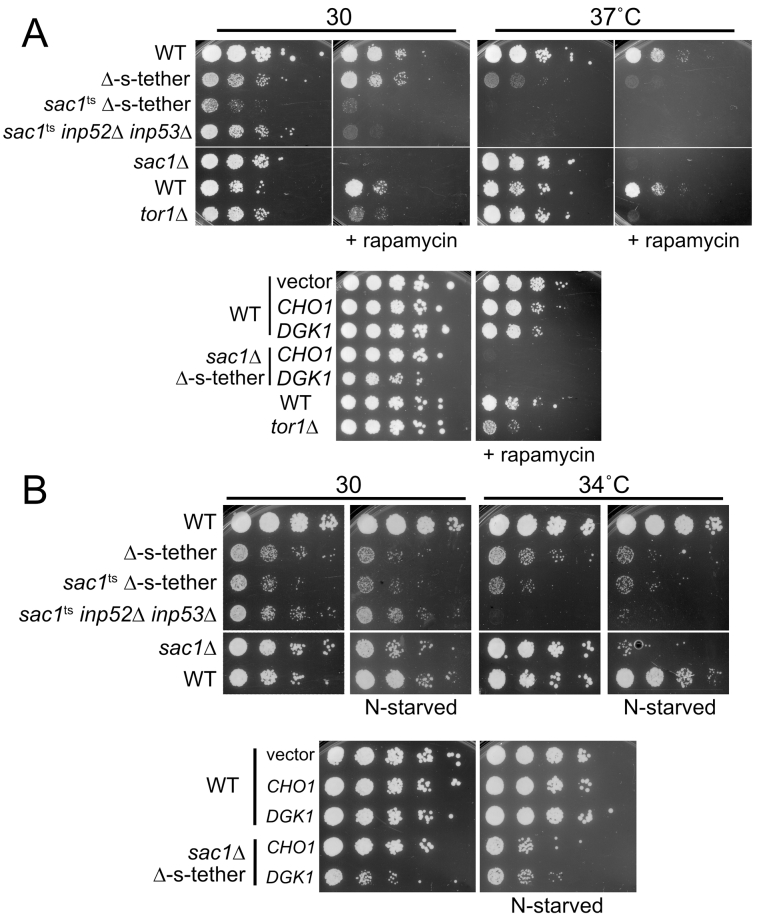
**Autophagy-dependent growth in response to *SAC1* and ER-PM tether elimination.***A*, (*Top*) Growth of ten-fold serial dilutions of Δ-s-tether (CBY5838), *sac1*^ts^*inp52*Δ *inp53*Δ (AAY143), *sac1*^ts^ Δ-s-tether (CBY6345) cells relative to their congenic WT strain (SEY6210). As shown, *tor1*Δ (CBY7612) and *sac1*Δ controls (CBY2809) with their congenic WT (BY4741) strain. Strains were cultured on solid medium containing 1 mM rapamycin and incubated for 4 to 5 days at 30 or 37 °C, as indicated. (*Bottom*) Ten-fold serial dilutions of *sac1*Δ Δ-s-tether cells transformed with *DGK1* and *CHO1* multicopy suppressors (CBY6508 and CBY6522) compared to their congenic WT strain transformed with the multicopy *DGK1* (pCB1346) and *CHO1* (pCB1352) plasmids. As the control for rapamycin sensitivity, dilutions of *tor1*Δ cells are shown relative to its congenic WT strain. *B*, (*Top*) Ten-fold serial dilutions of Δ-s-tether (CBY5838), *sac1*^ts^*inp52*Δ *inp53*Δ (AAY143), *sac1*^ts^ Δ-s-tether (CBY6345) cells with matched congenic WT strain (SEY6210), and *sac1*Δ cells (CBY2809) with its congenic WT (BY4741) strain. (*Bottom*) Ten-fold serial dilutions of WT and *sac1*Δ Δ-s-tether cells transformed with *DGK1* and *CHO1* multicopy suppressor plasmids. All strains were cultured in rich or nitrogen-depleted (N-starved) liquid media at the indicated temperatures, spotted onto solid growth media, and then incubated at 30 °C for 3 to 5 days.

To test if autophagy or its regulation by TORC1 is disrupted in *sac1*^ts^ Δ-s-tether and *sac1*^ts^ *inp52*Δ *inp53*Δ cells, these strains were cultured on growth medium containing the TOR kinase inhibitor rapamycin and on nitrogen-depleted medium. Although WT cell growth is unaffected by treatment with 1 nM rapamycin, *sac1*^ts^ Δ-s-tether and *sac1*^ts^ *inp52*Δ *inp53*Δ cells are sensitive to 1 nM rapamycin at 30 °C, similar to the rapamycin-sensitive *tor1*Δ control strain (Fig. S8*A*). Despite that *sac1*Δ had a nominal effect on autophagy gene expression (Fig. 10*B*), *sac1*Δ cell growth is also sensitive to rapamycin (Fig. S8*A*), likely due to the critical role of Sac1p in autophagosome–lysosome fusion (91). When cells are nitrogen starved, growth is inhibited in autophagy mutants due to the decrease in intracellular free amino acid pools that limits protein synthesis (92). After incubating at 30 °C or 34 °C for 3 days in nitrogen-depleted liquid medium, cell cultures were spotted onto rich solid medium to assess recovery from nitrogen starvation. Although Δ-s-tether cells show minor growth defects after nitrogen starvation, *sac1*^ts^ Δ-s-tether cells are marginally sensitive as compared with growth in nitrogen-replete medium at 30 or 34 °C (Fig. S8*B*). At 30 °C, *sac1*^ts^ *inp52*Δ *inp53*Δ cell growth is not affected by nitrogen starvation and *sac1*^ts^ *inp52*Δ *inp53*Δ cells do not grow at 34 °C regardless of culture condition (Fig. S8*B*). At 34 °C, the growth of *sac1*Δ cells is inhibited after culturing in nitrogen-depleted medium (Fig. S8*B*). In general, autophagy-related growth defects of *sac1*^ts^ Δ-s-tether and *sac1*^ts^ *inp52*Δ *inp53*Δ cells are consistent with the transcriptomics data. The results suggest that an autophagy regulatory response is elicited when *SAC1* is inactivated in cells already affected by PI4P dysregulation.

In *sac1*^ts^ Δ-s-tether cells, specific transcriptional changes are also observed that suggest a unique signature of synergistic responses due to the combination of mutations in *SAC1* and ER-PM tethers (Figs. 10 and S6). In *sac1*^ts^ Δ-s-tether cells incubated at 37 °C for 1 h, 480 genes are uniquely downregulated and 230 genes are uniquely upregulated (Fig. S6*A*). KEGG pathway analysis of these uniquely affected genes indicates that they represent ribosome, ribosome biogenesis, and other metabolic pathways, consistent with synergistic ESR defects that are otherwise less apparent in *sac1*^ts^ *inp52*Δ *inp53*Δ cells or Δ-s-tether cells (Fig. S6*B*).

### High-copy suppressors partly restore normal gene expression to *sac1*Δ Δ-s-tether cells

**Supporting Figure S9:**
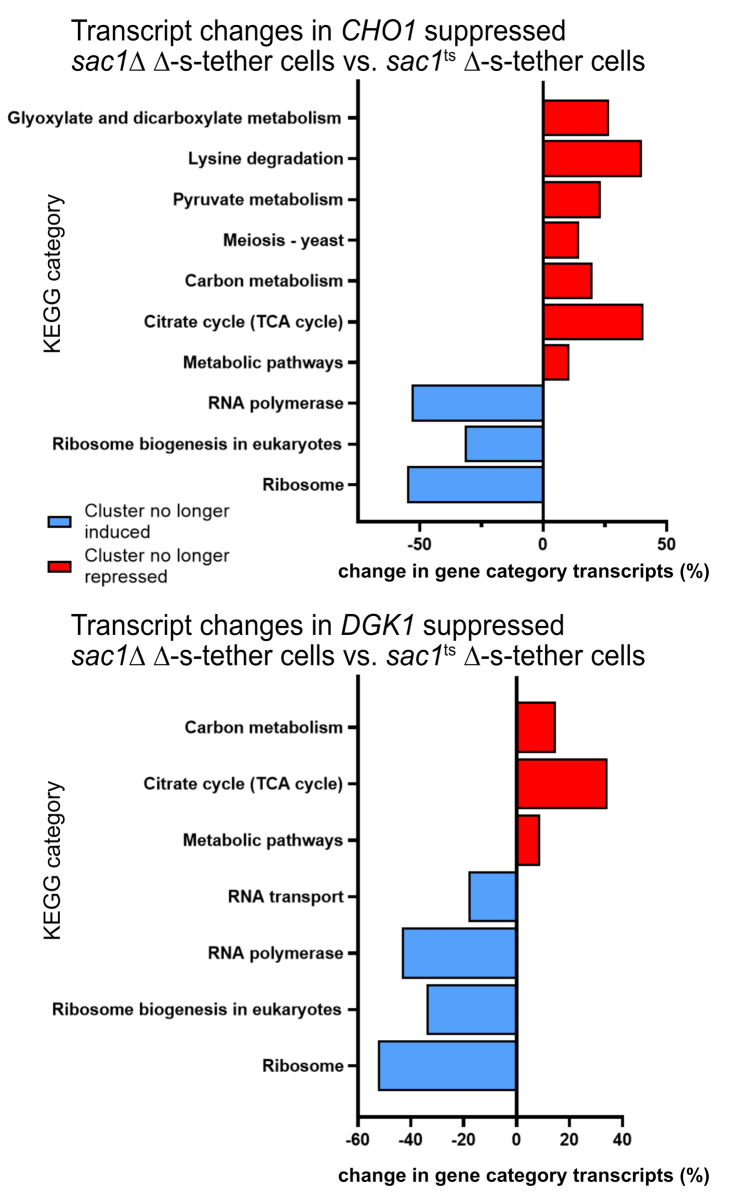
**KEGG analysis of transcriptomic changes in *CHO1*- and *DGK1*-suppressed *sac1***^**ts**^**Δ-s-tether cells.** Gene expression changes listed by KEGG category comparing *sac1*^ts^ Δ-s-tether (CBY6345) transcript levels at 37 °C for 1 h with the *CHO1-* and *DGK1*-suppressed *sac1*^ts^ Δ-s-tether cells (CBY6522 and CBY6508). Most significant changes in category transcripts are indicated that correlate with the suppression of *sac1* Δ-s-tether lethality.

In total, RNA-seq analysis revealed 948 upregulated and 839 downregulated genes in *sac1*^ts^ Δ-s-tether cells, relative to WT. Transcriptome analysis of *DGK1-*supressed *sac1*Δ Δ-s-tether cells shows only 677 upregulated and 443 downregulated genes, and *CHO1-*suppressed strains have 585 upregulated and 417 downregulated genes, indicating a partial restoration of the WT genomic expression profile. Specifically, KEGG analysis indicated that *DGK1* and *CHO1* suppression resulted in a normalization of general metabolism and ribosome biogenesis transcript levels (Fig. S9). These categories generally represented genes uniquely affected in *sac1*^*ts*^ Δ-s-tether cells, as opposed to Δ-s-tether or *sac1*^*ts*^ *inp52*Δ *inp53*Δ cells (Fig. S6*B*).

*DGK1* or *CHO1* suppression of *sac1*Δ Δ-s-tether lethality also correlates with a partial mitigation of ESR gene expression (Fig. 11). High-copy *DGK1* and *CHO1* increases and restores rESR gene expression in *sac1*Δ Δ-s-tether cells to WT levels, although iESR gene expression is marginally affected by the suppressors (Fig. 11). As predicted, phospholipid biosynthetic gene expression is no longer induced in suppressed *sac1*Δ Δ-s-tether cells, which correlates with partial dampening of the rESR response, even though iESR gene expression is still elicited (Figs. 11 and S3*B*). Presumably this suppression is sufficient to reduce the overwhelming level of cellular stress that otherwise results in lethality.

As validation of the genomic responses detected by RNA-seq, we showed that *DGK1* and *CHO1* suppressors confer growth resistance to *sac1*Δ Δ-s-tether cells when challenged with ER stress (Fig. S5*B*). Although *sac1*^ts^ Δ-s-tether cells are inviable in the presence of 3 mM DTT regardless of temperature, multicopy *DGK1* or *CHO1* suppresses the lethality of *sac1*Δ Δ-s-tether and confers robust growth on DTT-containing solid medium (Fig. S5). By alleviating phospholipid defects in *sac1*^ts^ Δ-s-tether cells, *DGK1* or *CHO1* overexpression appears to protect against membrane stresses that elicit ESR.

Unlike membrane stresses, however, multicopy *DGK1* or *CHO1* does not suppress the growth defects of *sac1*Δ Δ-s-tether cells when challenged with 1 nM rapamycin treatment at 30 °C (Fig. S8*A*). Moreover, when compared with growth in rich medium, *sac1*Δ Δ-s-tether cells expressing *DGK1* and *CHO1* multicopy plasmids do not fully recover after culturing in nitrogen-depleted medium for 3 days at 30 °C (Fig. S8*B*). Although multicopy *DGK1* or *CHO1* rescues *sac1*Δ Δ-s-tether lethality, it does not confer suppression to autophagy-related defects. As such, phosphoinositide dysregulation caused by *SAC1* inactivation in Δ-s-tether cells seems to be the primary trigger of autophagy gene expression, and neither *DGK1* nor *CHO1* restores normal PI4P distribution (Fig. S2*B*). The autophagy defects are, however, independent of the essential overlapping role of *SAC1* and ER-PM tethers as suppressed by *DGK1* and *CHO1*.

## Discussion

ER-PM MCSs coordinately regulate lipid metabolism by acting as direct conduits for lipid transport (5, 6, 93, 94, 95). Through exchange of lipid precursors between membranes, phospholipids are generated in the mitochondria, ER, and PM where tether proteins interact with different lipid transfer proteins to mediate lipid transfer. Lipid exchange between membranes is driven by concentration gradients maintained by differences in lipid levels and intracellular distribution (96). The ER and PM concentration of PS and PI4P drives their counter-directional transport, which in yeast is partly regulated by Sac1p turnover of PI4P levels in the ER and the generation of PI4P by Stt4p in the PM (96). The lethality of *SAC1* deletion in Δ-s-tether cells represents the disruption of intersecting mechanisms promoting PS/PI4P exchange, namely, bringing the ER and PM into close proximity for facilitating intermembrane lipid transfer and PI4P hydrolysis in the ER membrane to generate a PI4P concentration gradient between the ER and PM. Similarly, PS levels in the ER also contribute to the PS/PI4P transport cycle, and we show the dependence of PS levels on the phospholipid flux initiating from DAG biosynthesis. We further report the interdependency of ER-PM MCS formation and lipid metabolic pathways that maintain PS/PI4P homeostasis. The elimination of multiple PI phosphate phosphatases (or to a lesser degree, Sac1p alone), or PI kinases, induces cER association with the PM *via* an increase in the membrane tether Tcb3p. Moreover, augmenting PS synthesis by increasing phospholipid flux through increased expression of specific phospholipid biosynthetic genes restores growth and partially rescues lipid imbalances in *sac1*^ts^ Δ-s-tether cells. As shown by transcriptomic comparisons, defects in *sac1*^ts^ Δ-s-tether and *sac1*^*ts*^ *inp52*Δ *inp53*Δ cells lead to the upregulation of membrane stress response pathways that most notably affect the ER, and also elicit the broader ESR pathway. Apart from membrane stress, autophagy gene expression is constitutively activated in *sac1*^ts^ Δ-s-tether and *sac1*^*ts*^ *inp52*Δ *inp53*Δ cells. The autophagy response is primarily attributable to phosphoinositide defects, as opposed to membrane defects caused by disruption of ER-PM MCSs. In general, these results underscore the reciprocal relationship between the formation of contact sites and lipid homeostasis, particularly with respect to phosphoinositide regulation and phospholipid synthetic flux between the ER and PM.

Although the lipidomic profiles of Δ-s-tether and *sac1*^*ts*^ Δ-s-tether cells indicate substantial increases in DAG and TAG levels at the expense of phospholipid synthesis, and DAG polarization in the PM was disrupted, PA distributions were unaffected in all mutants analyzed. The lethality of *sac1*^*ts*^ Δ-s-tether cells was suppressed by increasing the dosage of phospholipid biosynthetic genes (*ALE1*, *DGK1*, *CHO1* and *SLC1*) that promote DAG consumption in phospholipid generation (Fig. 1*A*). The disruption of phospholipid biosynthetic flux in Δ-s-tether cells is predicted to decrease PI levels, which would be compounded by Sac1p elimination that otherwise contributes to overall cellular PI *via* PI4P hydrolysis. It was somewhat unexpected then that high-copy *PIS1*, which generates PI from CDP-DAG (61), does not suppress *sac1*^*ts*^ Δ-s-tether lethality. Previous reports showed decreases in steady-state PI levels in *sac1*Δ cells (57), although in our hands PI levels are modestly elevated in *sac1*Δ but unaffected in *sac1*^ts^ *inp52*Δ *inp53*Δ cells (Fig. 3*A*). PI is also required for yeast complex inositol sphingolipids, and the mole percentage of inositol sphingolipids (IPC) is unaffected in *sac1*Δ but reduced in *sac1*^ts^ *inp52*Δ *inp53*Δ and *sac1*^ts^ Δ-s-tether cells (Fig. 3*C*) (47, 57). Suppressors of *sac1*Δ Δ-s-tether lethality, however, do not restore IPC levels (although ceramide levels are no longer reduced). The *CHO1* and *DGK1* suppressors do not appear to mitigate *sac1*Δ defects in PI production or sphingolipid regulation in the context of the SPOTS complex (63). These results point to another phospholipid, other than PI, as central to the overlapping functions of *SAC1* and ER-PM MCSs.

Although the lipidomic profiles of Δ-s-tether, *sac1*Δ, *sac1*^ts^ *inp52*Δ *inp53*Δ, and *sac1*^ts^ Δ-s-tether indicated metabolic defects in several phospholipid species, membrane distributions of DAG, PS, PI4P, and PI(4,5)P_2_ are the most perturbed in *sac1*^*ts*^ Δ-s-tether cells. CDP-DAG is also necessary for PS production *via* the synthase encoded by *CHO1*, which is a particularly effective suppressor of *sac1*^*ts*^ Δ-s-tether lethality. This result implicates PS metabolism in the overlapping function of *SAC1* and ER-PM MCSs. Consistent with this conclusion, PS homeostasis contributes to maintenance of DAG polarization in the PM (97), which was also disrupted in *sac1*^*ts*^ Δ-s-tether cells. Indeed, all suppressors of *sac1*Δ Δ-s-tether lethality affected PS and PI4P localization resulting in their partial redistribution, as well as restoration of PI(4,5)P_2_ distribution. Although the PS and PI4P distribution is not normal as compared with WT, these suppressors appear to reestablish a partial PS/PI4P balance between the PM and ER/internal membranes.

In WT cells, *DGK1* or *CHO1* overexpression causes an accumulation of cytoplasmic ER and, while *CHO1* overexpression increases cER-PM association, *DGK1* overexpression had little effect (Fig. 6) (50, 60, 65). We therefore tested if increases in nonspecific contact between cytoplasmic ER and the PM generally suppress *sac1*Δ Δ-s-tether lethality. *HMG1* overexpression, which causes cytoplasmic ER expansion, could not rescue *sac1*^ts^ Δ-s-tether cells indicating that increasing cytoplasmic ER does not restore contact between the ER and the PM (Fig. 6*F*). For *sac1*Δ Δ-s-tether suppression, the enzymatic function of Dgk1p and Cho1p is required, which excludes the possibility of any direct membrane tethering conveyed by either protein. Moreover, additional tethering provided by expression of the artificial ER-PM staple does not suppress *sac1*Δ Δ-s-tether cells growth defects, indicating that nonspecific ER-PM contact is insufficient (Fig. S1). In other words, the intersecting functions of Sac1p and ER-PM MCSs are dependent on the specific tethering otherwise eliminated in Δ-s-tether cells.

Like the elimination of yeast ORPs, the inactivation of *SAC1* combined with the deletion of other PI phosphate phosphatases induces Tcb3p-dependent ER-PM MCSs (50). When yeast ORP genes are deleted, Tcb3p expression increases causing further recruitment of cER to the PM (50). Increased Tcb3p expression and ER-PM association is also observed in response to ER and PM stresses including those induced by lipid dysregulation, such as sterol depletion (5, 50). As observed in *sac1*Δ and *sac1*^*ts*^ *inp52*Δ *inp53*Δ cells, the redistribution of PI4P and its accumulation in the PM was also predicted to incur membrane stress (Fig. 7) (98). As such, any perturbation in cellular PI4P levels or distribution might elicit ER or PM stresses that increase Tcb3p-dependent ER-PM MCSs, even those caused by *stt4*^ts^ and *pik1*^ts^ PI-4-kinase defects that impact different intracellular pools of PI4P (99). Combining mutations that eliminate ER-PM MCSs with mutations affecting key lipids regulating ER and PM integrity likely provokes an intolerable cellular stress.

Osh6p is a suppressor (albeit poor) of *sac1*^ts^ Δ-s-tether cells growth defects (Fig. 9). These genetic interactions are consistent with models in which the differential concentration of PI4P generated by Sac1p in the ER and Stt4p in the PM drives Osh6p lipid transfer at ER-PM MCSs (5, 75, 83, 84). We propose that increasing the flux of PA and phospholipid synthesis (through either *ALE1*, *CHO1*, *DGK1*, or *SLC1* overexpression) also fuels Osh6p-dependent PI4P/PS counter-exchange by producing PS in the ER. Even the partial reestablishment of PI4P distribution between the PM and ER through increased phospholipid flux rescues *sac1*Δ Δ-s-tether growth defects. The Osh protein family shares overlapping functions, but this mode of suppression appears to be Osh6p specific (80). Osh4p represents an Osh6p homologue involved in similar PI4P-dependent transfer between membranes, but Osh4p is primarily localized at the Golgi where it counter-exchanges PI4P for sterols, as opposed to PS (84). As such, *OSH4* overexpression did not rescue *sac1*^ts^ Δ-s-tether lethality (Fig. 9).

From the RNA-seq analysis, *sac1*^ts^ Δ-s-tether cells exhibit a transcriptomic profile like other previously reported Δ-s-tether mutants (50). Given the impact of removing ER-PM tethers, it is not surprising that signature UPR genes are induced, indicating substantial ER stress. Combining *sac1*^ts^ and Δ-s-tether mutations magnifies these and other membrane stresses eliciting ESR transcriptional changes, like those observed when the yeast ORP *OSH4* is inactivated in Δ-s-tether cells (50). Like *sac1*^ts^ Δ-s-tether mutations, the lethality of *osh4*^ts^ Δ-s-tether cells is also rescued by *DGK1*, suggesting a related mechanism of suppression that ameliorates membrane defects (50). However, the combination of *SAC1* and ER-PM tether mutations elicits unique changes in gene expression. In *sac1*^ts^ Δ-s-tether cells, significant changes in autophagy gene expression are observed that correlate with an upregulation of autophagy activators and a downregulation of autophagy repressors. This distinctive aspect of the *sac1*^ts^ Δ-s-tether transcriptomic profile is also observed in *sac1*^ts^ *inp52*Δ *inp53*Δ cells but not in Δ-s-tether cells. These differences suggest that phosphoinositide defects trigger an autophagy response, as opposed to being caused by membrane effects resulting from ER-PM tether deletion. Previous reports showed that autophagosome fusion with the vacuole is dependent on *SAC1* and other related phosphoinositide phosphate phosphatases (91, 100). However, to our knowledge, these results are the first to show a phosphoinositide-dependent control of autophagy gene expression.

As a substrate for PI(4,5)P_2_ synthesis, PI4P in the PM needs to be tightly controlled to maintain the vital roles of PI(4,5)P_2_ in membrane trafficking, cytoskeletal organization, signaling cascades, and cell polarization (101). In *sac1*^ts^ Δ-s-tether cells, the perturbation of PI4P had the knock-on effect of disrupting PI(4,5)P_2_ distribution, as shown by the cytoplasmic accumulation of the GFP-2xPH(PLCδ) PI(4,5)P_2_ probe (Fig. 8). Presumably changes in PI(4,5)P_2_ accessibility impede GFP-2xPH(PLCδ) contact with the PM thereby diverting the probe into the cytoplasm. In *sac1*^ts^ Δ-s-tether cells, disruptions in PI(4,5)P_2_ distribution are somewhat consistent with the observed morphological defects in Δ-s-tether cells, which exhibit failures in bud growth and mother/daughter cell separation (5). Thus, an important axis controlling PI(4,5)P_2_ in the PM involves maintaining PI4P distribution between the ER and PM, as driven by PS metabolism and facilitated by Osh6p-dependent PI4P/PS exchange at ER-PM MCSs. This appears to be a generally conserved mechanism, given that similar events involving PI(4,5)P_2_ in mammalian cells involve ORP5/8 recruitment to ER-PM contact sites for PI4P and PS exchange (102).

## Experimental procedures

### Strains, plasmids, microbial techniques

Yeast strains and plasmids used in the study are listed in Tables S1 and S2, respectively. Throughout the experiments, yeast strains were cultured in Yeast extract-Peptone-Dextrose, synthetic minimal or synthetic complete medium at 30 °C, unless otherwise mentioned. To test growth defects of temperature conditional mutants, *sac1*^*ts*^ Δ-s-tether and *sac1*^*ts*^ *inp52Δ inp53Δ* cells and WT controls were cultured at permissive growth temperatures (30 °C unless otherwise stated) and shifted to 37 °C, as specified. Yeast growth on 3 mM DTT (Sigma-Aldrich Canada Co), or 0.1 and 1.0 nM rapamycin (Bioshop Canada Inc), on solid selective medium was assessed after 3 to 5 days. As described, autophagy induction by starvation was tested by culturing yeast in nitrogen-depleted liquid synthetic medium for 3 days (91) at the indicted temperatures, and then cultures were spotted onto solid rich synthetic medium and incubated at 30 °C for 3 to 4 days. Recovery from nitrogen starvation was assessed compared with strain growth after culturing in rich medium for 3 days. Growth plate assays represent ≥3 trials.

High-copy suppressors of *sac1*Δ Δ-s-tether lethality were tested by plasmid shuffle involving the transformation of *LEU2-*marked suppressor plasmids into *sac1*Δ Δ-s-tether cells containing *SCS2* on a *URA3*-marked plasmid. Bypass suppression of *sac1*Δ Δ-s-tether growth defects was then evaluated by selection against cells containing the *SCS2 URA3-*marked plasmid on solid synthetic medium containing 1 g/l 5′-fluoroorotic acid (5′-FOA) (Bioshop Canada). DNA cloning and bacterial and yeast transformations were performed using standard techniques (103, 104).

Given the inherent genetic instability of *sac1*^ts^ Δ-s-tether cells, this strain was maintained by culturing at 30 °C on synthetic medium supplemented with 1 mM choline (Sigma-Aldrich Canada) or transformed with a *URA3*-marked plasmid containing *SCS2.* For the latter, before use in growth assays or integrations/transformations, cells transformed with the *SCS2* plasmid are selected against on 5′-FOA medium. Before and after each transformation or growth assay, the temperature sensitivity of *sac1*^ts^ Δ-s-tether-derived cells is confirmed by growth inhibition at 37 °C.

To generate the plasmid expressing *E. coli dgkA* from the yeast P^*GAL1*^ promoter (pCB1435), a *Sac*I/*Kpn*I fragment containing the P^*GAL1*^-*dgkA* fusion gene was subcloned from pRS424GAL1pr-DGK (69) into the same restriction sites in pRS426. To generate the catalytically dead *cho1*^D127A^ mutation, site-directed mutagenesis was performed on pCB1351 containing the full ∼1.5-kb *CHO1* gene. With the incorporation of the altered codon, the *CHO1* gene was PCR amplified with the mutagenic TCCCCACTCCTTCTCAATGT forward primer and the GTGGTTGGCATAGGCAATCC reverse primer. The amplified mutant plasmid product (pCB1427) was enriched over the original template plasmid by *Dpn*I digestion, and the mutagenic change was confirmed by DNA sequencing.

### Fluorescence microscopy and live-cell imaging

Superresolution fluorescence microscopy was performed on a Zeiss LSM 880 confocal laser scanning microscope with an Airyscan superresolution GaAsP detector and 63×/1.4 oil immersion objective (Zeiss). All fluorophores were acquired using pixel dwell times at approximately 1.31 μsec per pixel. DsRed-HDEL fluorescence was excited using a 561-nm laser, and GFP fusions were excited using a 488-nm laser. Relative intensities were set to 1.5 for both lasers. Digital gain was set to 900 for the 488-nm laser and 800 for the 561-nm laser. Images were Airyscan processed in Zen Black and deconvolved in Zen Blue (Zeiss). ER association with the PM was determined by tracing the cell cortex in the Zen Blue profile mode, then measuring cER fluorescence intensity at the cortex. ER association with the cell cortex was expressed as a ratio of the total distance of cER fluorescence to the total PM perimeter. Images were exported as 8-bit uncompressed TIFF files then processed in Affinity Photo (Serif Ltd). Contrast enhancement was kept constant for each series of images. Levels of PM Lact-C2-GFP fluorescence were quantified using ImageJ (https://imagej.nih.gov/ij/index.html) by determining the mean fluorescence of selected areas corresponding to the cell cortex.

### Extragenic suppressor selection

To select for extragenic suppressors of *sac1*^ts^ Δ-s-tether lethality, a 2μ high-copy yeast genomic plasmid library was transformed into *sac1*^ts^ Δ-s-tether cells and cultured at 37 °C. To avoid isolation of ER-PM tether genes as dosage suppressors, genomic DNA was purified from Δ-s-tether (CBY5838) cells and partially digested using *Sau*3AI and 10-kb fragments were size selected from and ligated into the *Bam*HI digested 2μ vector, YEplac195. This 2μ genomic library was then transformed into *sac1*^ts^ Δ-s-tether cells and cultured on solid synthetic medium lacking leucine and uracil for 3 to 7 days at 37 °C. Surviving colonies from an equivalent of 9000 transformants (∼6 genomic equivalents) were selected and colony purified. Individual genomic library plasmids were recovered and retransformed back into *sac1*^ts^ Δ-s-tether cells to confirm suppression at 37 °C*.* Genes on each suppressor DNA fragment were identified by DNA sequencing. To identify individual suppressing genes from multiple genes on each genomic fragment, each candidate suppressor was individually cloned into YEplac195 and tested for suppression after transformation into *sac1*^ts^ Δ-s-tether cells. Bypass suppression was tested by subcloning suppressor genes into YEplac181, the suppressor constructs were then transformed into *sac1*Δ Δ-s-tether cells containing *SCS2 URA3*-marked plasmid to maintain viability, and then the *URA3* plasmid was counter-selected by streaking transformants onto solid selective synthetic medium containing 5′-FOA.

### Lipidomics

For lipidomics analysis, cells were first grown in culture to ∼0.8 *A*_600_ and pelleted cells were mixed with 200 μl methanol and 100 μl deionized water. Resuspended cell pellets were sonicated three times for 15 s on ice, after which 500 μl methanol and 200 μl of chloroform were added to each sample. The sonication was repeated before centrifugation at 21,000*g* for 15 min. The clarified supernatant was collected and dried under a gentle nitrogen gas flow at room temperature. As a normalization control, protein concentrations of resuspended cell pellets were determined by Bradford analysis (Sigma-Aldrich Canada). Dried supernatant residues were dissolved in 20 μl isopropanol per microgram of protein in the original resuspended cell pellet extracts.

Liquid chromatography (LC)-mass spectrometry (MS) lipid analyses from extracted, dried supernatants and resolubilized samples were performed at the University of Victoria Genome BC Proteomics Centre. For the analysis, a quality control sample was prepared by pooling 10 μl of each sample solution. For LC-MS analysis, 10-μl aliquots of sample solutions and the quality control sample were injected in a random order into a Thermo LTQ-Orbitrap Velos Pro mass spectrometer. Using 0.1% formic acid (A) and 0.1% formic acid acetonitrile–isopropanol 2:1 (B), samples were analyzed in duplicate on a C8 LC column (2.1 × 50 mm, 1.7 μm) at 50 °C with a flow rate of 0.4 ml/min as described (105). Samples were then analyzed by ultraperformance liquid chromatography–high-resolution MS using full-mass detection within *m/z* 200 to 1800 with (+) and (−) electrospray ionization. Along with full-mass MS runs, LC–tandem mass spectrometry determinations were acquired using collision-induced dissociation.

For the two full-mass LC-MS datasets acquired, raw data files were converted to a common data format and then processed in R using a customized script for peak detection, retention time, shift correction, peak grouping, and peak alignment in two rounds. Data processing provided *m/z* ratios, retention time, and peak area of the detected lipids in pairs. Raw data for both ESI+ and ESI- polarities were subject to principal component analysis with *m/z* ratios and retention time being set as x-variable markers. The peak area for each putative marker was normalized by the total markers area and used for quantification. Mass deisotoping and removal of chemical background peaks were then performed. Using HMDB (https://hmdb.ca/spectra/ms/search) and COMP-DB (www.lipidmaps.org/tools/ms/lm_mass_form.php) databases, lipid identities were assigned based on corresponding lipid *m/z* ratios; allowable mass error was set to ≤5 ppm in all cases. Lipids that presented a coefficient of variation ≥30% (12.8% of lipids) were excluded from further evaluation. For the (+) ion detection data, ionic forms of (M + H)+ and (M+Na)+ were used, whereas (M-H)-, (M+Na-2H)-, and (M+Cl)- forms were used for the (−) ion detection dataset. As described (106), *m/z* ratios were matched to specific lipids based on accurate mass matching, class-specific retention time, and adduct-type consistency. Values are represented as lipid content relative to WT.

### Phospholipid pulse labeling

Prior to pulse labeling of *de novo* synthesized phospholipids, WT and mutant cells were cultured at 30 °C to a density of *A*_600_ = 1.0 in synthetic growth medium. For temperature-sensitive conditional mutants, strains were then shifted to 37 °C for 1 h prior to labeling. To initiate phospholipid labeling, cells were cultured with 50 μCi/ml [^32^P]H_3_PO_4_ and equal samples were removed after 2, 5, 10, and 20 min of incubation. Labeling was terminated by resuspending cells in 5% trichloroacetic acid and then chilling on ice for 30 min prior to lipid extraction (107). Phospholipid extraction and lipid separation by one-dimensional paper thin-layer chromatography (TLC), was essentially performed as described (36). Before separation, samples were spotted and dried onto TLC silica gel 60 plates (MilliporeSigma), which were then imaged and analyzed on an Amersham Typhoon IP phosphoimager (GE Healthcare Life Sciences).

### Genomic expression analysis

Transcriptomic analysis by RNA-seq was performed as described (50). Mid-log-phase cells were cultured at 30 °C in synthetic minimal medium, or temperature-sensitive cells were cultured at 30 °C before incubation at 37 °C for 1 h, prior to poly(A) mRNA isolation and cDNA library generation. Read quality controls, alignments, read counting, gene ontology, and statistical analysis were performed as described (50). RNA-seq data were validated by quantitative PCR as described (50), whereby transcript levels were quantified by comparing *SIP18* and *KAR2* cDNA amounts with amplified *ACT1* cDNA as the internal control.

## Data availability

All data are contained within the article.

## Supporting information

This article contains supporting information (108, 109, 110, 111, 112, 113, 114, 115, 116, 117).

## Conflict of interest

The authors declare that they have no conflicts of interest with the contents of this article.
