# Supplementary material for: Increased Phospholipid Flux Bypasses Overlapping Essential Requirements for the Yeast Sac1p Phosphoinositide Phosphatase and ER-PM Membrane Contact Sites
Source: J Biol Chem. 2023 Jul 26;299(9):105092. doi: 10.1016/j.jbc.2023.105092 (PMC10470028; doi:10.1016/j.jbc.2023.105092)
Supplement: Supporting Tables S1 and S2 [file mmc1.docx]

**Supporting information**

**Supplemental Table 1. Yeast Strains.** Unless otherwise referenced, all strains were created as part of this study.

| Strain | Genotype | Source |
| --- | --- | --- |
| AAY143 | SEY6210 *inp52*Δ::*HIS3 inp53*Δ::*TRP1 sac1*Δ::*TRP1* [*sac1-23 LEU2 CEN*] | 109 |
| BY4741 | *MATa leu2*Δ*0 ura3*Δ*0 his3*Δ*0 met15*Δ*0* | 110 |
| CBY1048 | BY4741 *ire1*∆*::kanMX4* | 110 |
| CBY2809 | BY4741 *sac1*Δ::*kanMX4* | 110 |
| CBY5090 | BY4741 *stt4-4*^ts^:*kanMX4* | Gift from P. Stirling |
| CBY5092 | BY4741 *pik1-139*^ts^:*kanMX4* | Gift from P. Stirling |
| CBY5838 | SEY6210 *ist2*Δ::*hisMX6 scs2*Δ::*TRP1 scs22*Δ::*hisMX6 tcb1*Δ::*kanMX6 tcb2*Δ::*kanMX6 tcb3*Δ::*hisMX6 ice2*Δ::*natMX4* | 5 |
| CBY6146 | CBY5838 *sac1*Δ::*hphMX* [*SCS2 URA3 CEN*] |  |
| CBY6345 | CBY5838 *sac1*Δ::*hphMX* [*sac1-23 LEU2 CEN*] | 5 |
| CBY6508 | CBY5838 *sac1*Δ::*hphMX* [*DGK1 LEU2 CEN*] |  |
| CBY6522 | CBY5838 *sac1*Δ::*hphMX* [*CHO1 LEU2 CEN*] |  |
| CBY7553 | SEY6210 *cho1*∆::*kanMX4* |  |
| CBY7612 | BY4741 *tor1*∆::*kanMX4* | 110 |
| SEY6210 | *MAT*𝛼 *leu2‐3,112 ura3‐52 his3*Δ*200 trp1*Δ*901 lys2‐801 suc2*Δ*9* | 111 |

**Supplemental Table 2. Plasmids.** Unless otherwise referenced, all strains were created as part of this study.

| Plasmid | Description | Source |
| --- | --- | --- |
| pCB237 | *OSH6 URA3* 2µ | 112 |
| pCB241 | *OSH4 URA3* 2µ | 112 |
| pCB1167 | P*^ACT1^*-eGFP-P4M-SidM *URA3* *CEN* |  |
| pCB1185 | P*^ACT1^*-GFP-Myc-HMH-RitC *LEU2* *CEN* | 5 |
| pCB1345 | *PIS1 LEU2* 2µ |  |
| pCB1346 | *DGK1 LEU2* 2µ |  |
| pCB1350 | *SLC1 LEU2* 2µ |  |
| pCB1351 | *CHO1 URA3* 2µ |  |
| pCB1352 | *CHO1 LEU2* 2µ |  |
| pCB1382 | *ALE1 LEU2* 2µ |  |
| pCB1402 | *HMG1 URA3* 2µ |  |
| pCB1427 | *cho1*^D127A^ *URA3* 2µ |  |
| pCB1435 | P*^GAL1^*-*dgkA* *URA3* 2µ |  |
| Lact-C2-GFP-p416 | Lact-C2-GFP *URA3* *CEN* | 113 |
| pGPD416-C1δ-GFP | P^GPD^-C1δ-GFP *URA3* *CEN* | 68 |
| pRS416-DsRed-HDEL | DsRed-HDEL *URA3 CEN* | 114 |
| pRS424GAL1pr-DGK | P*^GAL1^*-*dgkA TRP1* 2µ | 69 |
| pRS426 | *URA3* 2µ | 115 |
| pRS426-G20 | P*^TEF2^*-GFP-Spo20[*51-91*] *URA3* 2µ | 69 |
| pRS426GFP-2xPH(PLCδ) | P*^PRC1^*-GFP-2xPH(PLCδ) *URA3* 2µ | 109 |
| pSCS2 | P*^PHO5^*-Myc-Scs2 *URA3* *CEN* | 116 |
| pWK092 | *TCB3*-eGFP *URA3* *CEN* | 117 |
| YCplac111 | *LEU2 CEN* | 118 |
| YCplac111 *sac1-23* | *sac1-23 CEN LEU2* | 109 |
| YEplac181 | *LEU2* 2µ | 118 |
| YEplac195 | *URA3* 2µ | 118 |
